# Responses of the *Emiliania huxleyi* Proteome to Ocean Acidification

**DOI:** 10.1371/journal.pone.0061868

**Published:** 2013-04-12

**Authors:** Bethan M. Jones, M. Debora Iglesias-Rodriguez, Paul J. Skipp, Richard J. Edwards, Mervyn J. Greaves, Jeremy R. Young, Henry Elderfield, C. David O'Connor

**Affiliations:** 1 Ocean and Earth Science, National Oceanography Centre Southampton, University of Southampton, Southampton, United Kingdom; 2 Institute for Life Sciences, University of Southampton, Southampton, United Kingdom; 3 Department of Ecology, Evolution and Marine Biology, University of California Santa Barbara, Santa Barbara, California, United States of America; 4 Centre for Proteomic Research, University of Southampton, Southampton, United Kingdom; 5 Centre for Biological Sciences, University of Southampton, Southampton, United Kingdom; 6 Department of Earth Sciences, University of Cambridge, Cambridge, United Kingdom; 7 Department of Earth Sciences, University College London, London, United Kingdom; 8 Department of Biological Sciences, Xi'an Jiaotong-Liverpool University, Suzhou, China; King Abdullah University of Science and Technology, Saudi Arabia

## Abstract

Ocean acidification due to rising atmospheric CO_2_ is expected to affect the physiology of important calcifying marine organisms, but the nature and magnitude of change is yet to be established. In coccolithophores, different species and strains display varying calcification responses to ocean acidification, but the underlying biochemical properties remain unknown. We employed an approach combining tandem mass-spectrometry with isobaric tagging (iTRAQ) and multiple database searching to identify proteins that were differentially expressed in cells of the marine coccolithophore species *Emiliania huxleyi* (strain NZEH) between two CO_2_ conditions: 395 (∼current day) and ∼1340 p.p.m.v. CO_2_. Cells exposed to the higher CO_2_ condition contained more cellular particulate inorganic carbon (CaCO_3_) and particulate organic nitrogen and carbon than those maintained in present-day conditions. These results are linked with the observation that cells grew slower under elevated CO_2_, indicating cell cycle disruption. Under high CO_2_ conditions, coccospheres were larger and cells possessed bigger coccoliths that did not show any signs of malformation compared to those from cells grown under present-day CO_2_ levels. No differences in calcification rate, particulate organic carbon production or cellular organic carbon: nitrogen ratios were observed. Results were not related to nutrient limitation or acclimation status of cells. At least 46 homologous protein groups from a variety of functional processes were quantified in these experiments, of which four (histones H2A, H3, H4 and a chloroplastic 30S ribosomal protein S7) showed down-regulation in all replicates exposed to high CO_2_, perhaps reflecting the decrease in growth rate. We present evidence of cellular stress responses but proteins associated with many key metabolic processes remained unaltered. Our results therefore suggest that this *E. huxleyi* strain possesses some acclimation mechanisms to tolerate future CO_2_ scenarios, although the observed decline in growth rate may be an overriding factor affecting the success of this ecotype in future oceans.

## Introduction

Anthropogenic increases in CO_2_ are expected to cause a reduction in ocean pH within the next century [1] with unknown consequences for marine organisms. Dissolution of CO_2_ in seawater causes the formation of carbonic acid (H_2_CO_3_), which dissociates to form bicarbonate (HCO_3_^−^) and H^+^ ions, causing a reduction in pH [2]. Liberated H^+^ ions react with carbonate ions (CO_3_^2−^), further increasing [HCO_3_^−^] whilst simultaneously causing a decrease in [CO_3_^2−^]. This reaction results in a drop in the saturation state of the mineral form of calcite (Ω-cal). When Ω-cal is <1.0, CaCO_3_ (hereafter referred to as calcite) becomes thermodynamically unstable and dissolution can exceed precipitation, resulting in CaCO_3_ loss. Ocean acidification is therefore expected to affect calcifying marine organisms that produce external CaCO_3_ structures such as shells, plates and exoskeletons [3], [4].

Coccolithophores, a group of unicellular eukaryotic phytoplankton with an external coating of biogenically precipitated CaCO_3_ plates (coccoliths), have been extensively studied in the context of ocean acidification [5]. This is because coccoliths are produced in intracellular compartments where Ω-cal >1.0 and CaCO_3_ saturation is tightly controlled. Coccoliths are subsequently extruded to the cell surface where they are subjected to the inorganic carbon chemistry conditions of surrounding seawater as modified by cell acid-base chemistry in the cell's diffusion boundary layer. Coccolithophores play a significant role in the biological carbon pump through the combined effects of photosynthesis and calcification, via the sedimentation of coccoliths, which are considered a major component of marine CaCO_3_ sediments [6], [7]. So far, only a limited number of studies have investigated the molecular mechanisms underlying the responses of coccolithophores to future CO_2_ scenarios, including a few mRNA-led studies [8], [9] and a recent microarray approach [10]. However, a poor correlation between protein and mRNA levels has often been found in mammalian, yeast and bacterial cells [11]–[15] and the coupling of protein and transcript abundance can be as low as 40% depending on the system [16]. Recent results suggesting an uncoupling between transcript and protein levels in coccolithophores under ocean acidification [8] also indicate that a proteomic approach is an appropriate method to define the functional responses of these species to changing CO_2_.

To investigate the proteomic response of coccolithophores to ocean acidification, cultures of *Emiliania huxleyi* (Lohmann) Hay et Mohler strain NZEH were grown under 395 and 1340 p.p.m.v. CO_2_, representing current day and projected CO_2_ levels for *circa* 2300 if all fossil fuel resources were released to the atmosphere [17]. We previously developed methods to enable quantitative proteomics on this species including a suite of bioinformatics tools to enable protein identification from a range of reference databases [18]. Therefore in addition to commonly measured physiological parameters such as cellular organic carbon and CaCO_3_, iTRAQ MS-based methods were applied to determine quantitative cellular proteomic differences between *p*CO_2_ conditions. iTRAQ tagging has recently been applied to other phytoplankton species, for example studies on light adaptation in marine cyanobacteria [19] and diatom toxicology [20] both utilize this approach as a means to investigate relative cellular proteome responses to environmental perturbations. For the current study, we used *E. huxleyi* NZEH, a heavy calcified strain, previously shown to exhibit an increase in calcification [21], [22] and a lower growth rate under high CO_2_ [21]. In view of a number of conflicting results previously reported for coccolithophore calcification under high CO_2_ [21]–[25], the aims of the present study were: (i) to extend the upper *p*CO_2_ level applied to *E. huxleyi* and examine its physiological responses, and (ii) survey the responses of its proteome to elevated CO_2_ levels. Extending the upper *p*CO_2_ level previously applied to NZEH [21] provided an opportunity to determine if our previous findings about calcification rates applied at worse case scenarios projected for CO_2_.

## Materials and Methods

### Culturing conditions

Oceanic seawater from the English Channel, offshore Plymouth (U.K.) was filtered through 0.22 µm Polycap capsules (Whatman, Maidstone, U.K.) and stored in the dark at 6°C. *Emiliania huxleyi* strain NZEH (also known as “PLY M219” and “CAWPO 6 A”) was obtained from the Plymouth Culture Collection of the Marine Biological Association of the U.K. This is a morphotype R strain that was isolated from the South Pacific in 1992. In all instances, cells were cultured in seawater supplemented with a modified *f* medium containing 100 µmol kg^−1^ nitrate, 6.24 µmol kg^−1^ phosphate and *f*/2 concentrations of trace metals and vitamins [26]–[28]. Cells were grown at 19.0±0.5°C in a 12∶12 h light: dark cycle typically between 80–120 µmol photons m^−1^ s^−1^ under Sylvania Standard F36W/135-T8 white fluorescent lighting (Havells Sylvania, Newhaven, U.K.). Cultures were bubbled with air containing either 385 or 1500 p.p.m.v. CO_2_ (BOC, Guildford, U.K.), filtered through 0.3 µm using sterile Hepavent discs (Whatman), which subsequently equilibrated to 395 and 1340 p.p.m.v. CO_2_.

Exponentially growing *E. huxleyi* cells were initially inoculated to a concentration of 7×10^3^ cells mL^−1^ into 2.4 L media that were previously bubbled for 72 h with the appropriate *p*CO_2_ in custom-built 3 L glass flasks fitted with Dreschel heads [21]. Cultures (<10^5^ cells mL^−1^) were bubbled very gently for ∼2–4 generations to allow cells to adjust to the bubbling regime. Aliquots of these cultures were inoculated into 20 L Nalgene polycarbonate culture vessels (Thermo Scientific Nalgene, Roskilde, Denmark) containing 14.7 L media to reach a starting cell density of 1.6×10^3^ cells mL^−1^. To enable dissolved inorganic carbon (DIC) equilibration, media within these vessels were bubbled for 7 days prior to the inoculation of cells. Cultures were continuously bubbled and, approximately 5–6 generations after inoculation (when cells were acclimated and cell density was ∼5×10^4^ to 10^5^ mL^−1^), samples were taken (start of experiment [“*t_1_”*] was day 6 and 7 for cells grown under 395 and 1340 p.p.m.v. CO_2_ respectively). Cells from this acclimation culture were used to inoculate a final 14.7 L culture to a starting concentration of 5×10^3^ cells mL^−1^. Cells were harvested after ∼4 generations, when cell density was ≤1×10^5^ cells mL^−1^ (time of harvest [“*t_2_”*] was day 8 for cells grown under 395 p.p.m.v. CO_2_ and day 9 or 10 for those grown under 1340 p.p.m.v. CO_2_). The treatment-dependent differences in sample days for *t_1_* and *_2_* relate to differences in growth rates between the two CO_2_ conditions. Experiments were conducted in triplicate and one flask for each air-CO_2_ mixture was left without cells to act as a blank for each experiment. Daily pH readings were taken using a calibrated pH meter to monitor changes through the experiment.

### Coccosphere volume, cell density and growth rate

Cell density and coccosphere (cells and their surrounding coccoliths) volume was measured daily. Aliquots of cultures were diluted 1∶10 with 3% NaCl (w/v) and analyzed using a Beckman Coulter Counter III fitted with a 70 µm aperture (Beckman Coulter, High Wycombe, UK). Mean coccosphere volume and cell density were calculated from triplicate samples and the growth rate (*μ*) was calculated as follows:
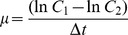
whereby *C*_1_ and *C*_0_ refer to the end and initial cell densities during the exponential phase of growth and Δ*t* refers to the duration of this phase. Following derivations from Novick and Szilard [29], the number of generations of growth (*g*) for each stage of the experiment was defined according to:
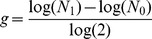
whereby *N*_1_ and *N*_0_ refer to the end and inoculum cell densities respectively.

### Particulate organic carbon and nitrogen

Samples for particulate organic carbon (POC) and nitrogen (PON) were obtained in triplicate at *t_1_* and *_2_* by filtration of culture aliquots onto pre-combusted 25 mm GF/F filters (Whatman). PIC was removed by acidification of the filters for 48 hours in a desiccator saturated with sulphuric acid fumes. Filters were then dried, pelleted and analyzed as previously described [14]. Cellular POC production (P_POC_, pmol POC cell ^−1^ day ^−1^) was calculated according as follows:
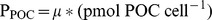



### Particulate inorganic carbon

Samples for cellular particulate inorganic carbon (specifically CaCO_3_) were obtained as previously described [21]. Coccolith-derived CaCO_3_ was dissolved by placing the filters in ∼20 mL 0.1 M HNO_3_, and [Ca^2+^] was determined using a Varian Vista Pro inductively coupled plasma emission spectrophotometer (ICP-OES; Agilent, Stockport, UK). Instrument calibration followed procedures used for the determination of Mg/Ca in foraminiferal calcite [30]. [Na^+^] was measured as a proxy for residual seawater Ca^2+^. Filters rinsed with the control media were also analyzed to confirm that the seawater contribution to measured [Ca^2+^] was negligible. Following the conversion of measured [Ca^2+^] to cellular CaCO_3_, production (P_CaCO3_, pmol CaCO_3_ cell ^−1^ day ^−1^) was calculated according as follows:
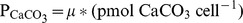



### Seawater nitrate, phosphate and silicate

Aliquots of 25 mL of culture were filtered through 0.22 µM for determination of nitrate, phosphate and silicate concentrations using a Seal QuAATro Autoanalyzer (Seal Analytical, Fareham, U.K.) with appropriate standards in the bracket of the expected concentrations.

### Dissolved inorganic carbon and total alkalinity

Culture aliquots were sampled for DIC and total alkalinity (TA) as previously described [21] and analyzed using the Versatile INstrument for Determination of Total Alkalinity (VINDTA; Marianda, Germany), a combined DIC and TA measuring system. Certified reference materials to calibrate and establish correction factors for VINDTA measurements were obtained from Professor Andrew Dickson at the Marine Physics Laboratory of the Scripps Institute of Oceanography, University of California San Diego, U.S.A. VINDTA-derived values for TA and DIC were corrected for various parameters including titration acid density, the nutrient concentration of the sample, temperature, salinity and reference material values. Final carbonate system parameters and pH were obtained with CO2SYS software [31] using a total pH scale (mol/kg-SW), K1 and K2 constants [32] with refits [33] and the acidity constant of the ion HSO_4_^−^ in sea water taken into account [34].

### Scanning electron microscopy and coccolith morphometrics

Samples for scanning electron microscopy (SEM) were pipetted onto 25 mm Isopore polycarbonate filters (Millipore), washed three times with alkaline milli-Q water (pH∼9) and dried at 37°C overnight. Filters were coated with a gold-palladium alloy and microscopic analysis was undertaken at the Natural History Museum, London, UK using a Zeiss Gemini Ultra Plus scanning electron microscope (Carl Zeiss, Oberkochen, Germany). The ImageJ software suite [35] was used to measure coccolith distal shield length and central area width [36]. A minimum of 23 detached coccoliths was considered for each replicate at *t_2_*. In total, 115 and 82 coccoliths were analyzed from cultures bubbled with 395 and 1340 p.p.m.v. CO_2_ respectively.

### Photochemical quantum efficiency

Maximum quantum efficiency of photosystem II (PSII) was analyzed at both *t_1_* and *_2_* using a Satlantic FIRe (Fluorescence Induction and RElaxation) system (Satlantic, Halifax, Canada) [37]. Measurements of *Fv/Fm* were taken four hours into the 12 h light phase, after samples were kept in the dark for 5 min.

### Protein extraction

At *t_2_*, cells were spun down at ∼500–1,000 *g* for 5 min and washed four times with calcium-free artificial seawater (pH 8) followed by hypersonication in 100 mM triethylammonium bicarbonate (pH 8.0) with 0.1% (w/v) SDS [18]. Proteins were subsequently precipitated using acetone (1∶10) and solubilized as previously described [18]. Protein concentrations in each sample were then assayed using the bicinchoninic method (Sigma Aldrich, Gillingham, U.K.).

### 8plex iTRAQ peptide labeling

Proteins were reduced, alkylated and then digested by adding 10 µL of 1 mg/mL trypsin in 80 mM CaCl_2_ followed by an overnight incubation at 37°C. The resulting trypsin digests were lyophilized and 90 µg of each sample were labeled with 8plex iTRAQ tags according to manufacturer's instructions (Applied Biosystems, Foster City, U.S.A.). iTRAQ tags 113, 115, 117 and 119 were added to peptides extracted from first, second, third and fourth 395 p.p.m.v. CO_2_ replicates respectively. Tags 114, 116, 118 and 121 were added to peptides from the first, second, third and fourth 1340 p.p.m.v. CO_2_ replicates. Protein digests were incubated at room temperature for 2 hours and vigorously vortexed because of the presence of a heavy CaCO_3_-like pellet. Resuspensions were spun down at 21,250 *g* for 30 min and supernatants were combined, dried overnight and stored at 4**°**C prior to tandem mass spectrometry (MS/MS) analysis. Data from the fourth replicate (reporter ions 119 and 121) were analyzed but excluded from further investigation because cell density thresholds of 10^5^ cells mL^−1^ were exceeded.

### 2D-UPLC and MS/MS

Two-dimensional separations were performed using a nanoAcquity 2D high performance liquid chromatography (UPLC) system (Waters, Elstree, UK) as described in **Methods S1**. All data were acquired using a Q-Tof Global Ultima (Waters, U.K.) and peak lists were generated as previously described [18]. MS/MS spectra were processed using a normal background subtraction with a 25% background threshold and medium de-isotoping with a threshold of 1%. No smoothing was performed. All spectra (24,119) obtained in these experiments were converted using PRIDE Converter [38] and are available in the PRIDE database [39] (www.ebi.ac.uk/pride) under accession number 20984.

### Protein identification and quantification

Peak lists from the MS/MS analysis were submitted to the Mascot search engine version 2.2.1 (Matrix Science, London, UK) against a database containing 130,000 *E. huxleyi* expressed sequence tags (ESTs) obtained from the NCBI EST database [40]–[43]. Searches were performed in all 6 EST reading frames and results were processed using BUDAPEST [18]. Output files from BUDAPEST analysis can be found in D**ata** **S1** and at http://bioware.soton.ac.uk/research/ehux/jones2012/

Additional analyses were performed against the *E. huxleyi* CCMP1516 draft genome produced by the US Department of Energy Joint Genome Institute (JGI; http://www.jgi.doe.gov) in collaboration with the user community. Searches were conducted against a non-redundant set of *E. huxleyi* CCMP1516 draft genome proteins using the “Emihu1_all_proteins.fasta” file downloaded on 02/16/12, which originally contained 217,034 filtered sequences from all models generated by the JGI Annotation Pipeline. This file was reduced to 115,995 non-redundant sequences for search purposes. Further searches were also conducted against a taxonomically restricted database constructed from UniProtKB sequences as described [18] with the addition of Chlorophyta. Mascot search criteria can be found in **Methods S1**. Pfam domain searches (*e*≤1.00e-04) [44] were conducted for any sequences that either lacked BLAST homology or only had homology to “hypothetical”, “predicted” or “unknown” proteins. Because of identification redundancy relating to the use of multiple search databases, we clustered identifications into Homologous Protein Groups (HPGs) using a combination of alignment and BLAST-based comparative analyses (similarity threshold of *e*≤1.00e-10 and >5% identity) [45].

Protein expression ratios were calculated using Mascot software, whereby peptide ratios were weighted and median normalization was performed, automatic outlier removal was selected and the peptide threshold was set to ‘at least homology’. If a protein identification only contained data for a single iTRAQ associated peptide, it was not further considered. Additionally, if an iTRAQ ratio was derived from only 2 peptides and one of these showed erroneously high or negative values, the protein was no longer considered for quantitative analysis. Geometric means, *G*, for the ratios of protein expression from each individual high: current day CO_2_ incubation were calculated and variance assessed by determination of the 95% confidence interval (*CI*). If the *G*±*CI* was ≥1.5, the protein was considered up-regulated and if the value was ≤0.67 then it was classed as down-regulated.

To expand our knowledge of the *E. huxleyi* proteome, data generated from the current study were combined with proteins identified from our previous proteomic investigation of *E. huxleyi* [18]. A combination of direct (accession number), indirect (protein name) and BLAST-based homology enabled the compilation of identifications from both studies. Results can be found in **Data S2**.

### Statistical analyses

We performed unpaired *t*-tests on physiological data using Sigma Stat 3.5 (Systat software, Hounslow, UK).

## Results

### Carbonate chemistry and culturing conditions

Measurements of DIC and TA were conducted at various times of the experiment and were analyzed by the VINDTA system. The remaining carbonate chemistry parameters (CO_3_^2−^, HCO_3_^−^, CO_2_, pH, and Ω-cal) were calculated (**Table 1**). At the time of cell harvest (*t_2_*), the media of cultures grown under 395.0 p.p.m.v. CO_2_ had a pH of 7.94 and an Ω-cal of 3.96 whilst cells grown under ∼1340 p.p.m.v. CO_2_ were in media with a pH of 7.47 and an Ω-cal of 1.52 (**Table 1**). At *t_2_*, less than ∼5% DIC was utilized by cells under 395 and 1340 p.p.m.v. CO_2_ compared to the blank, thus indicating that a semi-constant chemistry state was maintained throughout these experiments by keeping cell densities<10^5^ cells mL^−1^. Data from the fourth incubation were excluded from the analyses because cell densities exceeded this value. Carbonate chemistry values for *t_1_* and bubbled media prior to cell addition can be found in **Tables 1** **, S1** and **S2**.

**Table 1:** Mean carbonate chemistry values associated with experimental cultures (*t2*).

		Ambient	1340 p.p.m.v. CO_2_
***p*** **CO_2_ (p.p.m.v.)**	Initial^a^	433.1(395.9)^b^^,^ c	1398.4 (1376.8)
	End^d^	395.0 (397.6)	1340.6 (1412.7)
**[CO_2_] (µmol kg SW^−1^)**	Initial	14.4 (13.2)	47 (46.3)
	End	13.2 (13.4)	44.7 (47.7)
**[CO_3_^2−^] (µmol kg SW^−1^)**	Initial	167.4 (172.4)	65.6 (66.4)
	End	166.7 (174.2)	63.8 (64.3)
**[HCO_3_^−^] (µmol kg SW^−1^)**	Initial	1870.9 (1863.4)	2136.5 (2132.9)
	End	1785.5 (1856.3)	2037.1 (2140.6)
**[DIC] (µmol kg SW^−1^)**	Initial	2052.8 (2049.2)	2249 (2245.5)
	End	1965.4 (2043.8)	2145.7 (2252.6)
**Ω-cal**	Initial	3.97 (4.16)	1.57 (1.59)
	End	3.96 (4.18)	1.52 (1.55)
**pH**	Initial	7.92 (8.05)	7.48 (7.48)
	End	7.94 (7.95)	7.47 (7.48)
**TA (µmol kg SW^−1^)**	Initital	2292.4 (2293.2)	2304.2 (2302.5)
	End	2206.5 (2291.0)	2200.4 (2302.8)

a Average values at the beginning of the experiment before the inoculation of cells.

b Figures in parentheses represent blank seawater medium bubbled with the appropriate *p*CO_2_ mixture.

c There was only one sample available for the initial pre-inoculation blank of the ambient/current day CO_2_ condition. For this instance alone, values in parentheses are from the second 395 p.p.m.v. CO_2_ treatment.

d Average values at the end of the experiment (*t2*).

Cells were nutrient-replete throughout the entire experiment and, at the end of the experiment (*t_2_*), nitrate values ranged from 56.7–103.8 µmol kg^−1^ and phosphate values ranged from 2.4–4.2 µmol kg^−1^ (**Tables S3, S4**). At the end of the experiment (*t_2_*), there was no difference in nitrate utilization [*t*(4) = −0.0263; *p* = 0.980] between the low and high CO_2_ conditions, with respective decreases of 23.2%±4.7 and 23.3%±4.7. Also, even though there was a trend towards increased phosphate utilization under high CO_2_ at *t_2_*, this was not statistically significant [*t*(4) = −1.830; *p* = 0.141]. Cells incubated under 395 p.p.m.v. CO_2_ used 29.4%±6.3 of phosphate compared to 41.7%±9.8 for cells grown under the 1340 p.p.m.v. CO_2_ treatment.

### Cellular physiology

At the time of harvest (*t_2_*), substantial differences in cell physiology were observed for cells incubated under 1340 p.p.m.v. CO_2_. There was a significant increase in the cellular content of POC [*t*(4) = −3.233; *p* = 0.032], CaCO_3_ [*t*(4) = −7.015; *p* = 0.002] and PON [*t*(4) = −4.399; *p* = 0.012] at the end of the experiment and a significantly lower growth rate [*μ*; t(4) = 3.556; *p* = 0.024; **Table 2**]. However, no differences were encountered in CaCO_3_ [*t*(4) = −0.173; *p* = 0.871] and POC production rates [*t*(4) = −0.662; *p* = 0.544] or in CaCO_3_:POC [*t*(4) = 0.672; *p* = 0.538] and POC:PON [*t*(4) = 0.836; *p* = 0.450] cellular ratios. Similarly, there was no change in the photosynthetic energy conversion efficiency of PSII, with *Fv/Fm* values of 0.64±0.01 and 0.65±0.01 for cells incubated under 395 and 1340 p.p.m.v. CO_2_ respectively [*t*(4) = −0.164; *p* = 0.878; **Table S5**].

**Table 2:** Physiological parameters of *Emiliania huxleyi* NZEH grown under 395 and 1340 p.p.m.v. CO_2_ at *t1* and *2*.

Condition	*μ*	pmol POC cell^−1^	pmol POC cell^−1^ day^−1^
395 *t1*	1.63±0.36	0.87±0.03** b	1.43±0.35
1340 *t1*	1.30±0.23	1.43±0.15**	1.87±0.48
395 *t2*	1.29±0.04*	1.37±0.13* b	1.76±0.12
1340 *t2*	1.05±0.11*	1.84±0.22*	1.92±0.40

Condition	pmol CaCO_3_ cell^−1^	pmol CaCO_3_ cell^−1^ day^−1^	pmol cell^−1^ CaCO_3_:POC
395 *t1*	0.66±0.15^b^	1.09±0.44	0.75±0.15
1340 *t1*	0.98±0.17^a^	1.25±0.11	0.69±0.14
395 *t2*	1.09±0.05** b	1.41±0.10	0.81±0.10
1340 *t2*	1.37±0.04** a	1.44±0.20	0.75±0.09

Condition	pmol PON cell^−1^	pmol cell^−1^ POC:PON (C:N)	
395 *t1*	0.10±0.00*** b	8.52±0.15	
1340 *t1*	0.17±0.01***	8.58±0.54	
395 *t2*	0.15±0.01* b	9.28±0.49	
1340 *t2*	0.21±0.02*	8.82±0.92	

*, **, *** Significant results from t-tests comparing results from different *p*CO_2_ treatments at the same time point (i.e. 395 v 1340 p.p.m.v. CO_2_ at *t1*; 395 v. 1340 p.p.m.v. CO_2_ at *t2*) are designated as follows: ^*^ p≤0.05; ^**^ p≤0.01; ^***^ p≤0.001.

a, b, Significant results from t-tests comparing results from the same *p*CO_2_ treatment at different time points (e.g. 395 *t1* v 395 p.p.m.v. CO_2_ *t2*) are defined according to: ^a^ p≤0.05; ^b^ p≤0.01.

We observed differences in physiology between cells at different stages of the experiment (*t_1_* and *t_2_*). For example, there was a significantly higher cellular POC [*t*(4) = −6.385; *p* = 0.003] and PON content [*t*(4) = −5.790; *p* = 0.004] at *t_2_* compared to *t_1_* for cells grown under 395 p.p.m.v. CO_2_. Similarly, there was also a significantly higher cellular CaCO_3_ content at *t_2_* for cells incubated under both 395 [*t*(4) = −4.735; *p* = 0.009] and 1340 p.p.m.v. CO_2_ [*t*(4) = −3.927; *p* = 0.017; **Table 2**].

**Figure 1:**
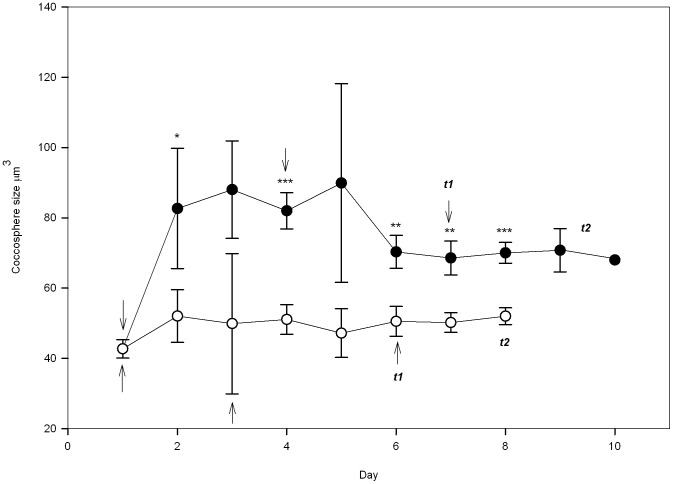
Comparison of *Emiliania huxleyi* NZEH coccosphere sizes at 395 and 1340 p.p.m.v. CO_2_. * p≤0.05; ** p≤0.01; *** p≤0.001. A point with no star indicates differences were non-significant. Arrows indicate inoculations into media with different *p*CO_2_ conditions, as outlined in Materials and Methods. Open circles represent cells grown under under 395 p.p.m.v. CO_2_ that were harvested after 12–13 generations (*t2* = day 8). Solid circles indicate cells grown under 1340 p.p.m.v. CO_2_. In order to ensure suitable biomass for proteomics, these were harvested after 9–12 generations (*t2* = day 9 or 10) because of their lower growth rates.

At the end of the experiment, increases in cellular CaCO_3_, POC and PON in cells grown under 1340 p.p.m.v. CO_2_ coincided with an increase in coccosphere volume (**Figure 1**). Cells grown in nutrient-replete media equilibrated to this high CO_2_ condition showed an increase in coccosphere volume 24 h after inoculation, with a mean volume of 82.7 µm^3^±17.1 compared to 52.0 µm^3^±7.5 under 395 p.p.m.v. CO_2_. The difference in coccosphere volume between conditions was not always significant (**Figure 1**). However, as generational time increased and cells acclimated, the variability in coccosphere volume decreased (**Figure 1**). Consequently, coccosphere volumes at the time of harvest were significantly different, with a mean value of 69.44 µm^3^±8.37 for cells grown under the high CO_2_ treatment compared to 52.00 µm^3^±2.26 for cells grown under 395 p.p.m.v. CO_2_ [*t*(4) = −3.465; *p* = 0.026].

**Figure 2:**
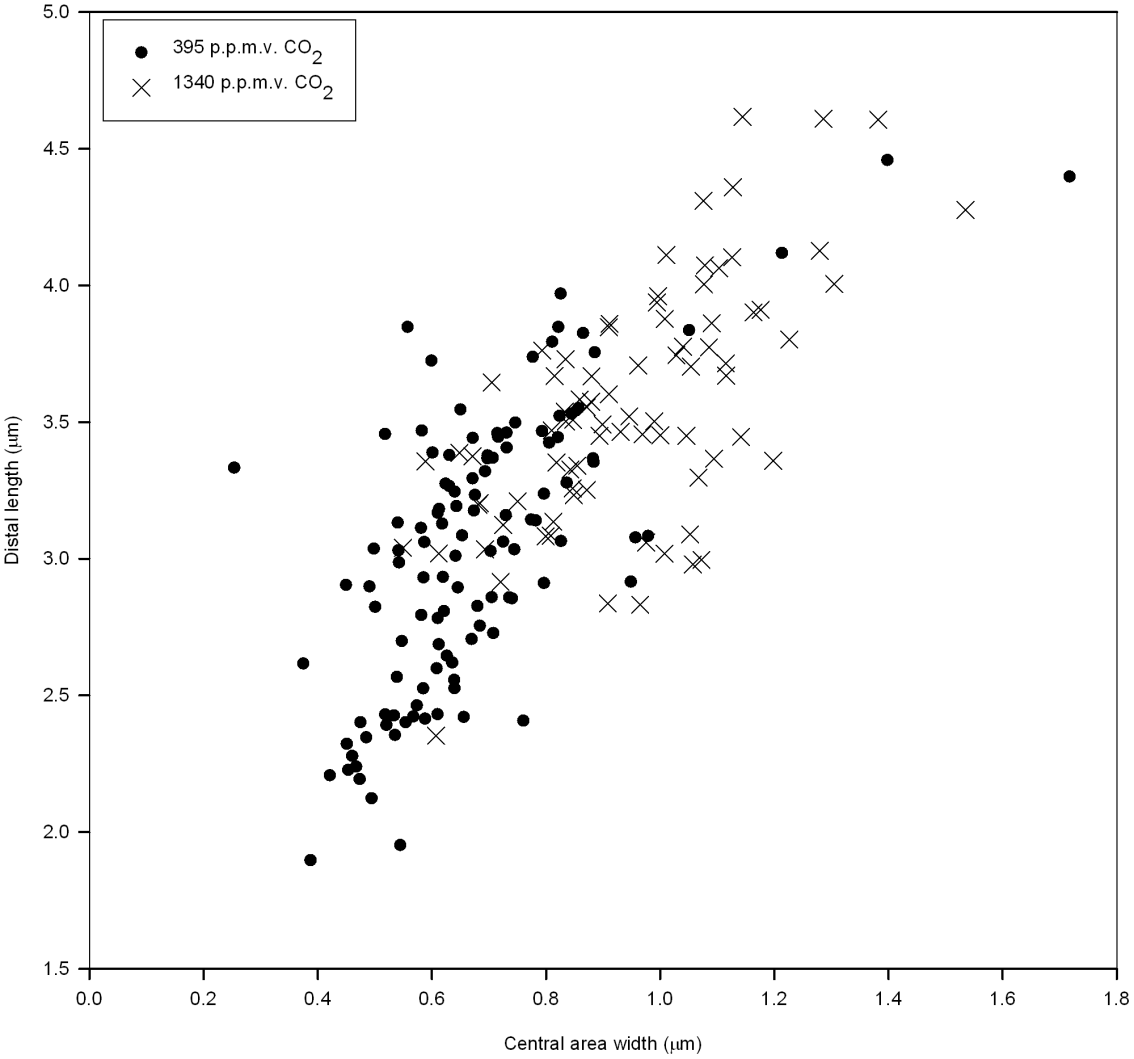
Morphometric analysis of detached coccoliths obtained at the end of the experiment (*t2*) from monoclonal cultures of *Emiliania huxleyi* NZEH bubbled with 395 and 1340 p.p.m.v. CO_2._

**Figure 3:**
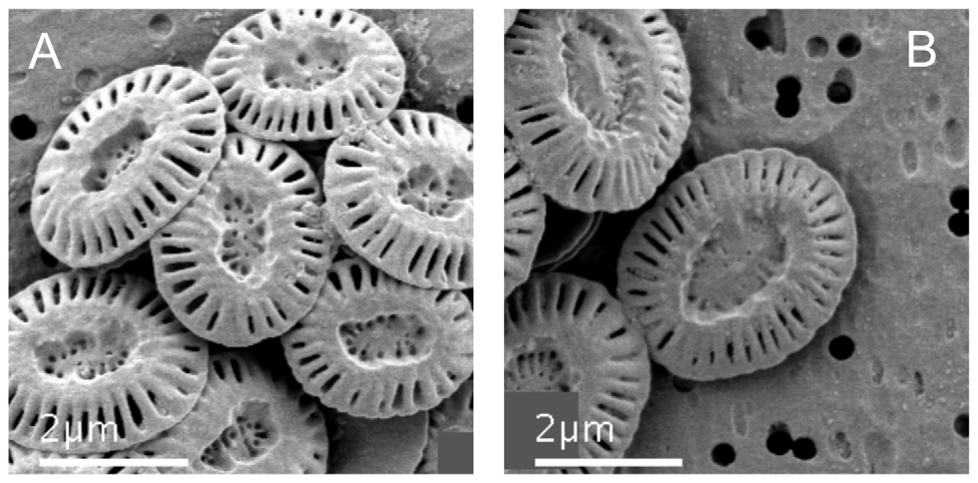
Example of coccoliths derived at the end of the experiment (*t2*). Panel A: typical coccoliths from 395 p.p.m.v. CO_2_ treatment; B: coccoliths from the lower pH (∼7.6) and 1340 p.p.m.v. CO_2_ treatment which are typically larger and are slighly less calcified but possess no signs of dissolution or malformation.

An increase in coccosphere volume at the time of harvest (*t_2_*) was accompanied by a significant increase in the size of individual coccoliths (**Figure 2**). Specifically, the mean distal length of coccoliths for cells grown under the high CO_2_ condition was 3.56 µm±0.44, whereas values for 395 p.p.m.v. CO_2_ were 3.03 µm±0.52 [*t*(195) = −7.526; *p*<0.001]. Coccoliths from cells incubated under 1340 p.p.m.v. CO_2_ did not show any sign of dissolution or malformation (**Figures 3a and 3b**). However, they were less calcified, possessing a central area width of 0.95 µm±0.19 compared to those from current conditions that had an average width of 0.67 µm±0.19 [*t*(195) = −10.080; *p*<0.001]. Despite these clear trends, we still found variation within the dataset that is typical for coccoliths derived from cultures.

### Proteomic identifications

MS/MS spectra of peptides derived from *E. huxleyi* protein extracts were searched against protein sequences from the *E. huxleyi* CCMP1516 draft genome. This yielded 40 matches from 37 homologous protein groups (HPGs) and the identification of 21 HPGs with iTRAQ quantification. Data were also searched against a taxonomically-restricted subset of UniProtKB. This resulted in the identification of 21 HPGs based on the assignment of at least two peptides, of which 10 could be quantified (**Tables 3** **and** **4**). Data were also searched against a database containing ∼130,000 *E. huxleyi* ESTs using Mascot, which resulted in 180 matches possessing ≥2 peptides. Following BUDAPEST analysis, 97 clusters were formed that comprised of 128 consensus sequences assembled from 150 ESTs. The manual removal of identifications with data for only one iTRAQ-tagged peptide resulted in 37 HPGs with quantification information (**Tables 3** **and** **4**). When all identifications from the genome project, the taxonomically restricted UniProtKB database and EST searches were considered and combined, we were able to identify 115 HPGs. Of these, 46 could be quantified; in depth information regarding all quantified and non-quantified identifications can be found in **Data S2**.

**Table 3:** Proteins (Homolgous Protein Groups) down-regulated in *Emilania huxleyi* NZEH under high CO_2_.

Identification	Accession^a^/EST cluster	114∶113^b^	116∶115	118∶117	Range^c^
30S ribosomal protein S7	Q4G343	0.28	0.45	0.61	0.27–0.66
Histone H2A	D0MWJ7	0.49	0.41	0.39	0.37–0.49
	87a	0.62	0.55	0.45	0.45–0.64
	EHUXJGI72235	0.49	0.45	0.39	0.38–0.50
Histone H3	EHUXJGI255477	0.36	0.20	0.32	0.20–0.40
Histone H4	C1MUM2	0.37	0.25	0.56	0.24–0.59
	92a	0.37	0.25	0.56	0.24–0.59
	EHUXJGI201707	0.37	0.22	0.39	0.21–0.46

a Identifications are associated with either UniProtKB accession, *Emiliania huxleyi* genome protein ID or BUDAPEST consensus sequence. Further information can be found in **Data S2;** BUDAPEST sequence files can be found in **Data S1**.

b Ratio of protein identified between the two CO_2_ treatments for each replicate incubation, whereby 114∶113 is the first replicate, 116∶115 is the second and 118∶117 the third. Reporter ions 114, 116 and 118 were applied to peptides extracted from the high CO_2_ treatments and 113, 115, 117 to the current day treatment.

c “Range” (*G*−*CI*)–(*G*+*CI*) as defined in Materials and Methods.

**Table 4:** Proteins (Homologous Protein Groups) exhibiting non-significant expressional change in *Emilania huxleyi* NZEH under high CO_2_.

Identification	Accession^a^/EST cluster	114∶113^b^	116∶115	118∶117	Range^c^
40S ribosomal protein S12	81a	2.09	2.01	0.27	0.28–3.90
	EHUXJGI240824	2.01	1.87	0.28	0.29–3.60
50S ribosomal protein L12 (chloroplastic)	86a	1.95	1.88	0.24	0.25–3.70
	EHUXJGI86748	1.86	1.83	0.23	0.24–3.60
Acyl carrier protein	97a	1.81	2.47	1.01	0.99–2.76^d^
Adenosylhomocysteinase	26a	0.27	1.05	1.27	0.27–1.86
Adenylate kinase (putative)	33a	1.45	1.82	0.68	0.67–2.18
Ankyrin repeat protein (putative)	51a	1.69	1.86	0.47	0.47–2.72
Apocytochrome f (precursor)	Q4G3D7	0.88	0.81	0.61	0.61–0.94
	35a	0.88	0.81	0.61	0.61–0.94
ATP ase/synthase (vacuolar)	91a	2.57	1.97	0.44	0.45–3.83
	EHUXJGI210241	2.48	1.88	0.45	0.45–3.62
ATP synthase subunit alpha	07a	0.71	0.96	1.24	0.69–1.30
	EHUXJGI199000	0.65	1.36	1.06	0.64–1.50
ATP synthase F(1) sector subunit alpha (chloroplastic)	Q4G397	0.32	0.78	1.12	0.32–1.35
	C7BEK1	0.40	0.70	1.11	0.38–1.20
ATP synthase F (0) sector subunit b′ (chloroplastic)	Q4G3A0	0.95	0.57	0.77	0.56–1.00
ATP synthase F(1) sector subunit beta (chloroplastic)	Q4G3C8	0.62	1.02	1.09	0.63–1.24
Chloroplast light harvesting protein isoform	12a	0.33	0.57	0.78	0.32–0.87^d^
	12b	0.24	0.44	0.90	0.22–0.96
Clathrin small chain (putative)	18a	1.21	1.36	0.84	0.83–1.48
Clp protease ATP binding subunit	Q4G3D0	0.43	0.59	0.74	0.42–0.78
Cold shock DNA-binding protein (putative)	96a	2.22	6.00	0.47	0.43–7.87
	EHUXJGI111020	1.23	1.32	0.70	0.71–1.55
Demethylmenaquinone methyltransferase (putative)	09a	1.98	1.91	0.40	0.41–3.23
ETC complex I protein (putative)	44a	1.29	1.25	0.73	0.73–1.51
Ferredoxin–NADP reductase	29a	0.59	0.90	0.79	0.58–0.96
FKBP-type peptidyl-prolyl cis-trans isomerase	50a	1.68	4.68	0.71	0.61–5.15
	50b	0.75	1.34	0.67	0.58–1.34
	EHUXJGI239227	1.58	3.20	0.76	0.70–3.53
Glycerol-3-phosphate dehydrogenase EC 1.1.1.8	45a	0.77	0.91	0.67	0.66–0.92^d^
	EHUXJGI87542	0.85	0.75	0.82	0.75–0.86
Histone H2B	89a	0.43	0.40	1.04	0.31–1.03
	EHUXJGI96192	0.43	0.52	0.89	0.38–0.90
Nascent polypetide-associated complex protein/transcription factor (putative)	52a	1.84	1.82	0.34	0.34–3.15
No BLAST hit	08a	0.76	0.69	0.32	0.32–0.94^d^
	EHUXJGI349877	0.82	0.67	0.32	0.32–0.99
No BLAST hit	13a	3.28	1.01	0.43	0.36–3.57
	EHUXJGI205571	2.51	1.01	0.53	0.45–2.67
No BLAST hit	57a	1.80	1.42	0.39	0.40–2.53
No BLAST hit	76a	1.16	1.27	0.76	0.76–1.42
	EHUXJGI237243	1.17	1.36	0.78	0.78–1.48
No BLAST hit	83a	1.90	1.70	0.42	0.42–2.87
	EHUX110219	1.32	1.35	0.87	0.88–1.53
No BLAST hit	EHUXJGI218259	3.12	2.52	0.31	0.31–5.73
Periplasmic binding/iron transport lipoprotein (putative)	EHUXJGI269712	1.16	0.95	1.23	0.95–1.29
Phosphoglycerate kinase	Q5ENR8	0.91	1.44	1.48	0.91–1.70
	11a	0.95	1.06	1.06	0.95–1.10^d^
	EHUXJGI270670	1.05	0.99	1.05	0.99–1.07
Phosphoribulokinase (chloroplastic)	14a	0.70	1.35	0.86	0.63–1.36^d^
Photosystem II 12 kDa extrinsic protein (chloroplastic)	12f	0.70	0.99	0.56	0.53–1.01
Photosystem II oxygen evolving enhancer (chloroplastic)	34a	1.29	1.09	0.73	0.73–1.40^d^
	27a	1.46	0.89	0.82	0.72–1.45^d^
	27b	1.38	0.84	0.83	0.71–1.37
	EHUXJGI265795	1.12	1.10	0.72	0.72–1.27
Predicted protein	65a	1.85	1.55	0.54	0.54–2.46
Predicted protein	82a	1.51	1.67	0.29	0.30–2.73^d^
Predicted protein	21a	0.98	0.91	0.72	0.72–1.04^d^
	21b	0.98	0.91	0.72	0.72–1.04
	21c	1.46	1.47	0.67	0.68–1.88
Predicted protein	72a	1.72	1.45	0.56	0.56–2.22
Predicted protein	84a	2.31	7.08	0.48	0.43–9.20
	EHUXJGI96453	2.22	7.28	0.47	0.41–9.32
	EHUXJGI243179	2.26	7.38	0.46	0.41–9.54
Ribulose bisphosphate carboxylase large chain	Q4G3F4	0.17	0.81	0.76	0.17–1.29
S-adenosylmethionine synthetase	06a	0.92	0.63	0.64	0.57–0.91
	06b	0.96	0.54	0.49	0.42–0.96
	06c	0.96	0.53	0.47	0.40–0.96
	EHUXJGI62235	0.91	0.85	0.51	0.51–1.05
	EHUXJGI267646	0.96	0.83	0.49	0.49–1.09
Ubiquitin family protein (putative)	01f	1.77	2.18	0.74	0.74–2.71

a, b, c As defined for table 3.

d If an identification was made from numerous ESTs with valid iTRAQ quantification, an geometric average has been made (see Materials and Methods). Please refer to **Data S2** for individual EST quantification.

### Proteomic correlates

Ratios of peptide abundance from each high: low CO_2_ replicate incubation were calculated (114∶113, 116∶115 and 118∶117) and values of *G*±*CI*≥1.5 or ≤0.67 used to determine up and down-regulation respectively (**Table 3**). According to these strict criteria, no HPGs were up-regulated in response to high CO_2_, while only four HPGs were down-regulated (histones H2A, H3 H4 and chloroplastic 30S ribosomal protein S7). When results from the *E. huxleyi* genome searches, protein databases and EST searches were combined, 43 HPGs showed no difference in regulation under high CO_2_ when all three replicates were considered. A number of proteins, however, showed trends of up- or down-regulation in the first two replicates, with a reduced/ambivalent response in the third replicate (**Table 4**).

## Discussion

### Physiological properties of *Emiliania huxleyi* NZEH under high CO_2_

This study extends our knowledge of the physiological response of *Emiliania huxleyi* strain NZEH (morphotype R) to increasing *p*CO_2_ through the use of traditional approaches and by quantitative proteomic analysis. Although previous studies have investigated the responses of this strain to varying CO_2_, our levels (1340 p.p.m.v.) were the highest applied so far, compared to 750 [14], [15] and 909 p.p.m.v. CO_2_ [46] used in previous studies.

In our study, cells were under nutrient repletion for the duration of the experiments (nitrate and phosphate levels at *t_1_* and *_2_* being between 86.18–103.79 and 3.23–4.32 µmol kg^−1^ respectively). Therefore, the observed increases in coccosphere volumes and cellular CaCO_3_, POC and PON content are not attributable to nutrient limitation. Using multiple generations of growth (∼9–12 and ∼12–13 generations for cells incubated under 1340 and 395 p.p.m.v. CO_2_ respectively), we found that multiple generations are a necessary step for the population to achieve acclimation despite some studies reporting acclimation after one or a few cell generations [47]. In our study, there were periods at the start of the acclimation phase when there was no difference in coccosphere size, but these trends were changed towards the end of the experiment, after multiple generations of growth during which the majority of cells had grown and divided under the experimental conditions. Calcification also changed, since cells contained significantly less cellular CaCO_3_ at the beginning of the experiment compared to after ∼5–8 generations (*t_2_*). Another example, in this case, showing the opposite trend, is growth rate, which displayed comparable values between the different treatments at start of the experiment, but declined after subsequent generations of incubation under 1340 p.p.m.v. CO_2_.

Observed increases in cellular POC content at elevated CO_2_ levels compared to current conditions are in agreement with other studies on strain NZEH [21], [22], [46]. Similarly, the observed increase in cellular CaCO_3_ is in agreement with some results using this strain [21], [22]. Despite an increase in CaCO_3_ content, coccosphere volume, and coccolith size under the high CO_2_ condition, coccoliths were slightly less calcified but with larger central area widths on the distal shield. However, there appeared to be no sign of coccolith malformation or dissolution under either *p*CO_2_ condition.

Cellular CaCO_3_:POC ratios did not change under high CO_2_, comparable to trends found by Iglesias-Rodriguez et al. [21] but in disagreement with findings by Hoppe et al. [46]. The CaCO_3_:POC ratio indicates the balance of CO_2_ fluxes as a result of calcification and photosynthesis, which appeared not to be altered under these “worst case” high (1340 p.p.m.v.) CO_2_ scenario. Furthermore, the CaCO_3_:POC ratio was <1.5 in all conditions tested, which means that the cells are likely to be a sink of CO_2_ to the surrounding environment [48]. CaCO_3_:POC ratios associated with 1340 p.p.m.v. CO_2_ were virtually identical to values reported in bubbling experiments under the highest *p*CO_2_ used on this strain by Iglesias-Rodriguez et al. [21] and by Hoppe et al. [46]. There was no difference in the cellular POC:PON ratio between the two CO_2_ conditions; this ratio was only 6.43% higher than previously reported for NZEH cells grown under 750 p.p.m.v. CO_2_ [21].

Cellular production rates of CaCO_3_ and POC did not vary significantly under 1340 p.p.m.v. CO_2_ compared to present-day conditions, unlike previous observations by Iglesias-Rodriguez et al. [21] and Shi et al. [22] who found increases in these parameters for this strain under 750 p.p.m.v. CO_2_. Hoppe et al. [46] also found an increase in POC production under elevated *p*CO_2_ but a decline in cellular CaCO_3_ production at 909 p.p.m.v. CO_2_. Since CaCO_3_ and POC production did not differ between treatments, the higher cellular CaCO_3_, PON and POC values that we found are related to the lower growth rate of cells incubated under 1340 p.p.m.v. CO_2_. At present, the source of the variability in carbon production under varying CO_2_ levels between our results and other studies on NZEH remains unresolved.

Changes in carbonate chemistry were not associated with any difference in the maximum quantum efficiency of PSII (as measured by *Fv/Fm*), a proxy of cellular photosynthetic health. This result was previously reported for cells of this strain incubated under 750 p.p.m.v. CO_2_ [21]. A decline in cellular growth rate (*μ*) under high CO_2_ is also in agreement with some investigations on this strain [21]; however others [22], [46] have found no change in growth under elevated *p*CO_2_. Eukaryote cells often undergo growth delay or arrest when threatened with damage to cellular components such as DNA [49] and significant growth rate reductions in the current study suggests that *E. huxleyi* cells are exhibiting one of the hallmarks of cellular stress response (CSR) to prevent damage to macromolecular components under high CO_2_ [50], [51].

### Proteomic responses of *Emiliania huxleyi* to ocean acidification

Four *E. huxleyi* HPGs from all three replicate experiments conducted under 1340 p.p.m.v. CO_2_ were down-regulated: histones H2A, H3 and H4 and a chloroplast located 30S ribosomal protein S7 (*rps7*). This is likely related to the lower rate of cell division observed under high CO_2_, which may be accompanied by a reduction in DNA and chromatin synthesis and a consequent decrease in the requirement for histone proteins [52]. Reduced *rps7* expression is also likely related to lower growth rates, although this has not been rigorously examined in phytoplankton [53]. Our results suggest that *E. huxleyi* cells grown under high CO_2_ undergo a reduction in translation initiation in some instances [54]. The non-differential expression of histone H2B under different CO_2_ conditions may be explained by indications that this protein is involved in processes other than nucleosome packaging since our identification the identified H2B from the *E. huxleyi* genome (protein ID 96192) also contained a 50S ribosome-binding GTPase (Pfam: CL0023) and a peptidase domain (Pfam: CL0012).

By nomenclature, we found that the expression of 43 HPGs (over 93% of those quantified) was unchanged in all three incubations under elevated *p*CO_2_. However, there was a tendency for some proteins to show differential expression within the first two of the three incubations (**Table 4**), which while not significant, is potentially indicative of sub-lethal CSR in *E. huxleyi* [50], [51]. For example, HPGs with homology to FKBP-type peptidyl-prolyl cis-trans isomerase and ubiquitin family proteins were substantially up-regulated within the first two high CO_2_ replicates (**Table 4**). Additionally a cold shock protein HPG was also up-regulated under high CO_2_ in the same two replicates; these proteins have roles in multiple stress responses [55]. Meanwhile, 40S and 50S ribosomal proteins were up-regulated almost twofold (**Table 4**) within these two high CO_2_ incubations, which might relate to induction of translational processes to replace damaged proteins or a role in extraribosomal functioning [56]. The potential up-regulation of an HPG containing an ankyrin repeat domain also suggests an increase in protein-protein interactions under high CO_2_ but it is difficult to decipher the role of this HPG because of the diverse functionality exhibited by proteins containing this domain [57]. Similarly, a trend for reduction of S-adenosylmethionine synthetase under high CO_2_ in the second and third incubations is difficult to discuss because of the multiple roles of this enzyme involved in methylation [58].

Trends from the first two incubations indicate that membrane related processes have the potential to be affected by high CO_2_. For example up-regulation of an acyl carrier protein from the first two incubations could be responsible for increased lipid production [59] that may be involved in membrane remodeling under low pH [60]. An up-regulation of vacuolar membrane bound V-ATPases in these two incubations could also relate to stress responses to cellular pH regulation under high CO_2_/low pH [61], [62]. However, none of these trends are significant and therefore they must be interpreted with caution.

Despite the disrupted cell cycle and some evidence for a proteomic CSR under high CO_2_, there are also indications of cellular tolerance to ocean acidification. We identified a broad range of HPGs whose expression remained unchanged (**Table 4**). These included processes such as electron transport (ETC complex I protein, apocytochrome f), photosynthesis (ferredoxin—NADP reductase, PSII 12KDa extrinsic protein, PSII oxygen evolving enhancer, various chloroplastic ATP synthase subunits), carbon fixation (phosphoribulokinase, RuBisCO large chain), glycolysis (phosphoglycerate kinase) and other metabolic processes (adenosylhomocysteinase). It should be noted that the lack of change in the relative levels of these proteins does not preclude effects on their activities due to the elevated *p*CO_2_ growth conditions. For example, it is well known that some of these central metabolic enzymes are regulated by post-translational modifications such protein phosphorylation. Collectively, however, the results suggest that this particular strain of *E. huxleyi* does not undergo major metabolic perturbations with respect to numerous photosynthetic and other metabolic processes once acclimated to high CO_2_ levels in these short-term experiments. Indeed, the down-regulation of a chloroplastic clp protease in the first two incubations (**Table 4**) suggests that *E. huxleyi* chloroplasts are not undergoing stress under high CO_2_.

### The unknown biochemistry of *Emiliania huxleyi*

Of the 115 quantified and non-quantified HPGs identified, 79 were not found within the current draft of the *E. huxleyi* genome (strain CCMP1516) and only 6 were found in the genome and both the EST and taxonomically restricted UniProt databases. This is likely a result of genomic variability within the *E. huxleyi* species group and we expect proteomic identifications to be assisted by the availability of sequences of other strains in the near future. However, it is worth noting that approximately 38% of quantified and non-quantified HPGs identified in the current study did not match any database homologue, Pfam conserved domain or only matched protein sequences defined as “unknown”, “predicted” or “hypothetical.” We also found unusual proteins, for example searches against the *E. huxleyi* genome uncovered an alanine-rich sulfotransferase protein (ID 270658) exhibiting homology to several mucin-associated surface proteins (MASPs) identified in the parasite *Trypanosoma cruzi* (**Data S2**). MASPs are members of a recently discovered gene family containing ∼1400 members that comprises ∼6% of the parasite diploid genome [63], [64] and are thought to have a role in host immune system evasion. Interestingly, we found homologs to this HPG in the *Thalassiosira pseudonana* genome (e.g. UniProtKB accessions B8BX85 and B8BTN5). At present, we are unable to propose a role for MASP-like proteins within marine eukaryotic algae.

The large number of highly expressed proteins with unknown features that we found within *E. huxleyi* highlights the multiple unknown biochemical facets of these species and illustrates that our incomplete knowledge on cellular processes, such as calcification. It is therefore likely that components other than those currently described for coccolithophores including proteins typically involved in carbon acquisition, proton extrusion or Ca^2+^ transport remain to be identified. For example, in two of our three high CO_2_ replicates, an HPG containing a structural motif found in demethylmenaquinone methyltransferases (DMMMs) was up-regulated. This was particularly interesting since DMMMs catalyze the last step of the biosynthesis of menaquinione (vitamin K2; MK) through the conversion of demenaquinone to MK, a pathway predominantly found within bacteria [65]. Within *E. huxleyi*, it is possible that this HPG could be involved in calcification since vitamin K is an essential cofactor of a γ-carboxylase that facilitates calcium ion chelation *via* the post-translational conversion of glutamic acid to γ-carboxyglutamyl residues. Through this process, vitamin K2 has been shown to regulate biomineralization by modulating the proliferation of osteoblastic cells [66]. Since similar DMMM proteins are present in a handful of other eukaryote phytoplankton such as *T. pseudonana* and *Phaeodactylum tricornutum*, MK could instead be an isoprenoid quinone within the photosynthetic or respiratory electron transport chains, as in some prokaryotes [67]–[69]. A further investigation of the role of this HPG and confirmation of its up-regulation in association with cellular high CO_2_ responses is therefore warranted.

To help develop future directions for research and assist the *E. huxleyi* genomics community, we combined data from the current study with those from our previous proteomic investigations of *E. huxleyi* [18] resulting in the experimental validation of 189 HPGs. There was limited overlap between the datasets from these two studies (26 HPGs), which is likely to reflect incomplete proteome sampling in each experiment as a result of the different protein extraction protocols and identification approaches.

### Could cellular tolerance lead to a competitive disadvantage in future oceans?

This study adds to the increasing body of literature highlighting variable responses in coccolithophore calcification relating to ocean acidification. For example, it has been recently shown that some coccolithophores may show increased [21], [22], [25] or negligible changes in calcification in response to increasing partial pressures of CO_2_ [9], [70]. Our results are particularly interesting in light of recent field data showing that heavily calcified *E. huxleyi* cells of the same morphotype (R) were associated with naturally low pH waters [71]. Another recent study found that *E. huxleyi* cells of another morphotype (A), different from that used in our study, possessed more CaCO_3_ in low pH waters [72]. Additionally, recent models propose that coccolithophores might become more heavily calcified and grow more slowly under high CO_2_ [73]. We argue that the strain of *E. huxleyi* used in this study exhibits reduced growth that may relate to cellular stress, suggested by the reduction in growth rate and the potential induction of a sub-lethal CSR proteome. This may, in part, explain these field results, since we found reduced cell division resulted in an increase in cellular CaCO_3_. The induction of shifts in the activities of these, and other pathways, may act as a trade-offs to enable the maintenance of cellular processes required in the short term, perhaps ensuring cellular homeostasis or remodelling during acclimation [51]. However, the decline in growth rates observed in our study could ultimately be disadvantageous for *E. huxleyi* under high CO_2_ [5] despite its ability to maintain many biological functions. This could be particularly detrimental in scenarios of phytoplankton group assemblages where other groups outnumber coccolithophores. For example some studies have shown that diatoms are able to maintain [22], [74] or even increase growth rates under increased *p*CO_2_ [75], [76], which could lead to increased interspecific competition and assemblage shifts in regions where coccolithophores currently dominate. Long-term investigations into coccolithophore CSRs are therefore required to confirm the extent to which they are responsible for cellular resilience to variations in pH and to determine the conditions under which cells are capable of adaptation to high CO_2_. This would elucidate whether a catastrophic metabolic collapse relating to acute stress occurs in this particular ecotype of *E. huxleyi* through time or if cellular mechanisms responding to high CO_2_ levels resulted in a steady recovery of growth rates, thus preventing outcompetition by other taxa that could otherwise cause a decrease in oceanic CaCO_3_ fluxes in a future high CO_2_ world.

## Supporting Information

Data S1 **Output files from BUDAPEST analysis.**
(ZIP)

Data S2 **Detailed information regarding proteins identified in the current study.**
(XLS)

Methods S1 **UPLC and MS/MS parameters; Mascot protein identification search parameters.**
(DOCX)

Table S1 **Mean carbonate chemistry parameters of 14.7L acclimation cultures before cell addition.**
(DOCX)

Table S2 **Mean carbonate chemistry parameters associated with 14.7L acclimation cultures at** ***t1*** **.**
(DOCX)

Table S3 **Nitrate, phosphate and silicate information for cells at** ***t1*** **.**
(DOCX)

Table S4 **Nitrate, phosphate and silicate information for cells at** ***t2*** **.**
(DOCX)

Table S5 ***Fv/Fm*** **values of PSII at** ***t2*** **.**
(DOCX)
