# Supplementary figures and images for: Responses of the Emiliania huxleyi Proteome to Ocean Acidification
Source: PLoS One. 2013 Apr 12;8(4):e61868. doi: 10.1371/journal.pone.0061868 (PMC3625171; doi:10.1371/journal.pone.0061868)

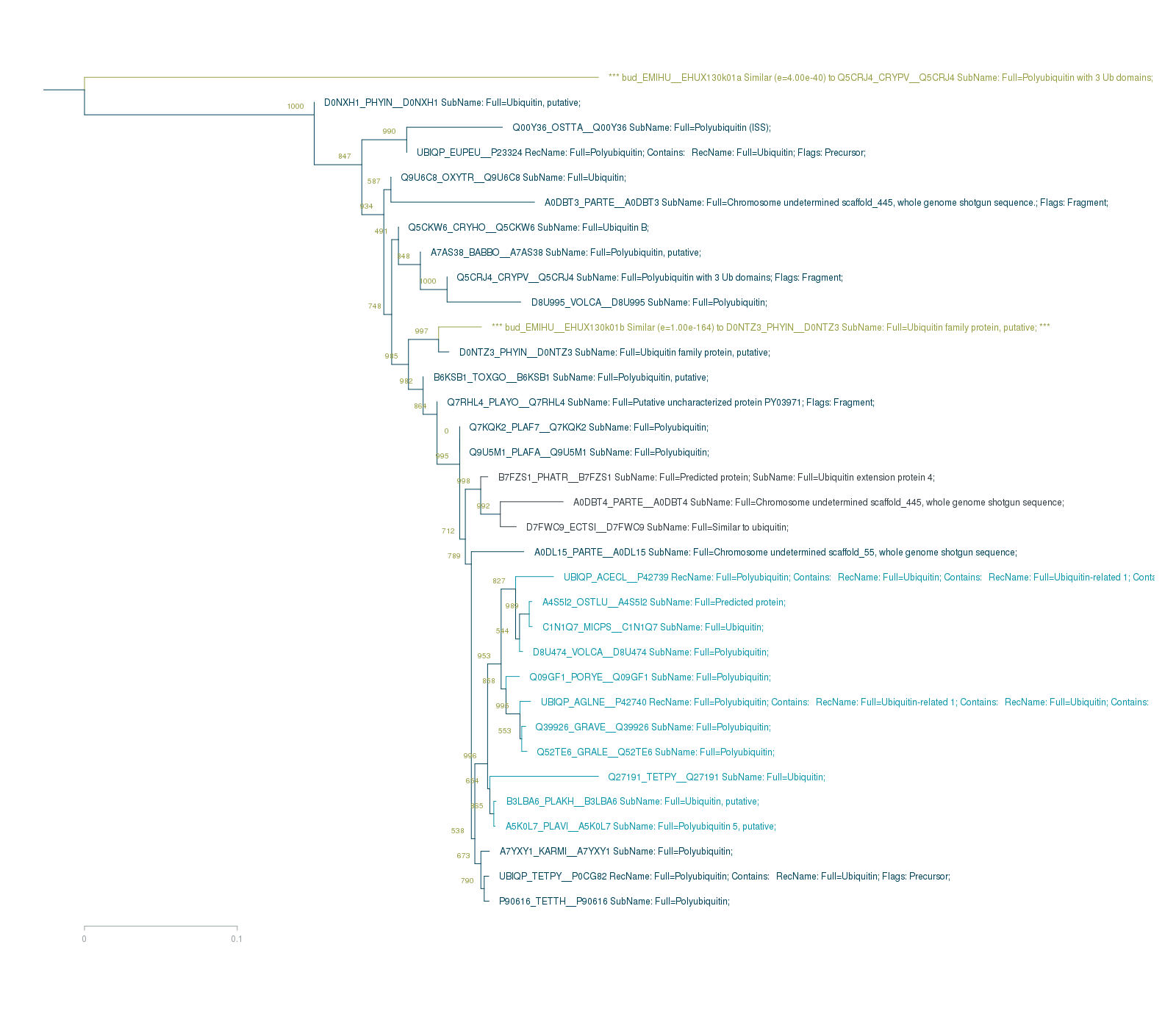

Supplement: Data S1 — Output files from BUDAPEST analysis. (ZIP) [file pone.0061868.s001.zip › BUDAPEST data/Jones_et_al_2012_Data/EHUX130k01a.png]

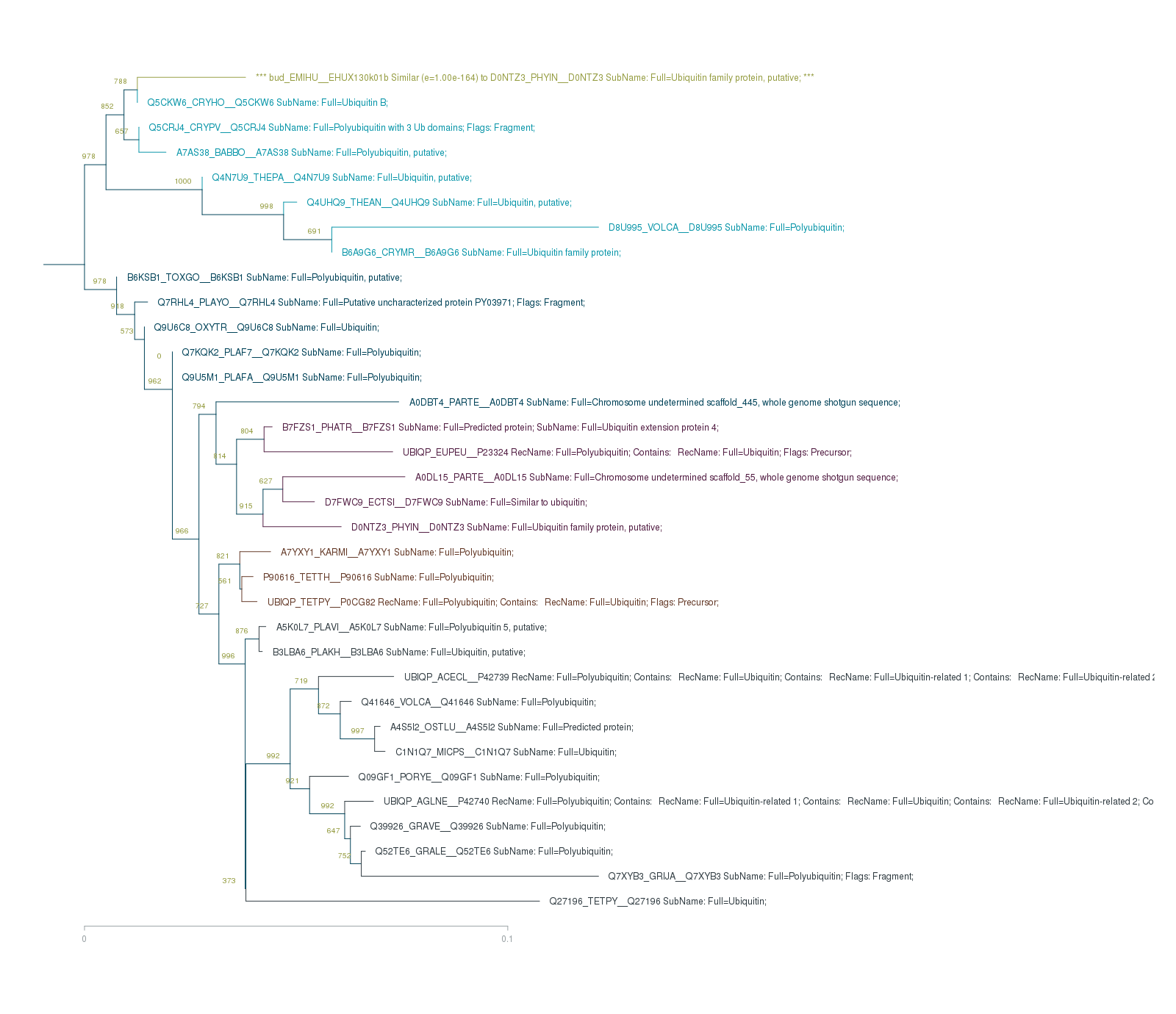

Supplement: Data S1 — Output files from BUDAPEST analysis. (ZIP) [file pone.0061868.s001.zip › BUDAPEST data/Jones_et_al_2012_Data/EHUX130k01b.png]

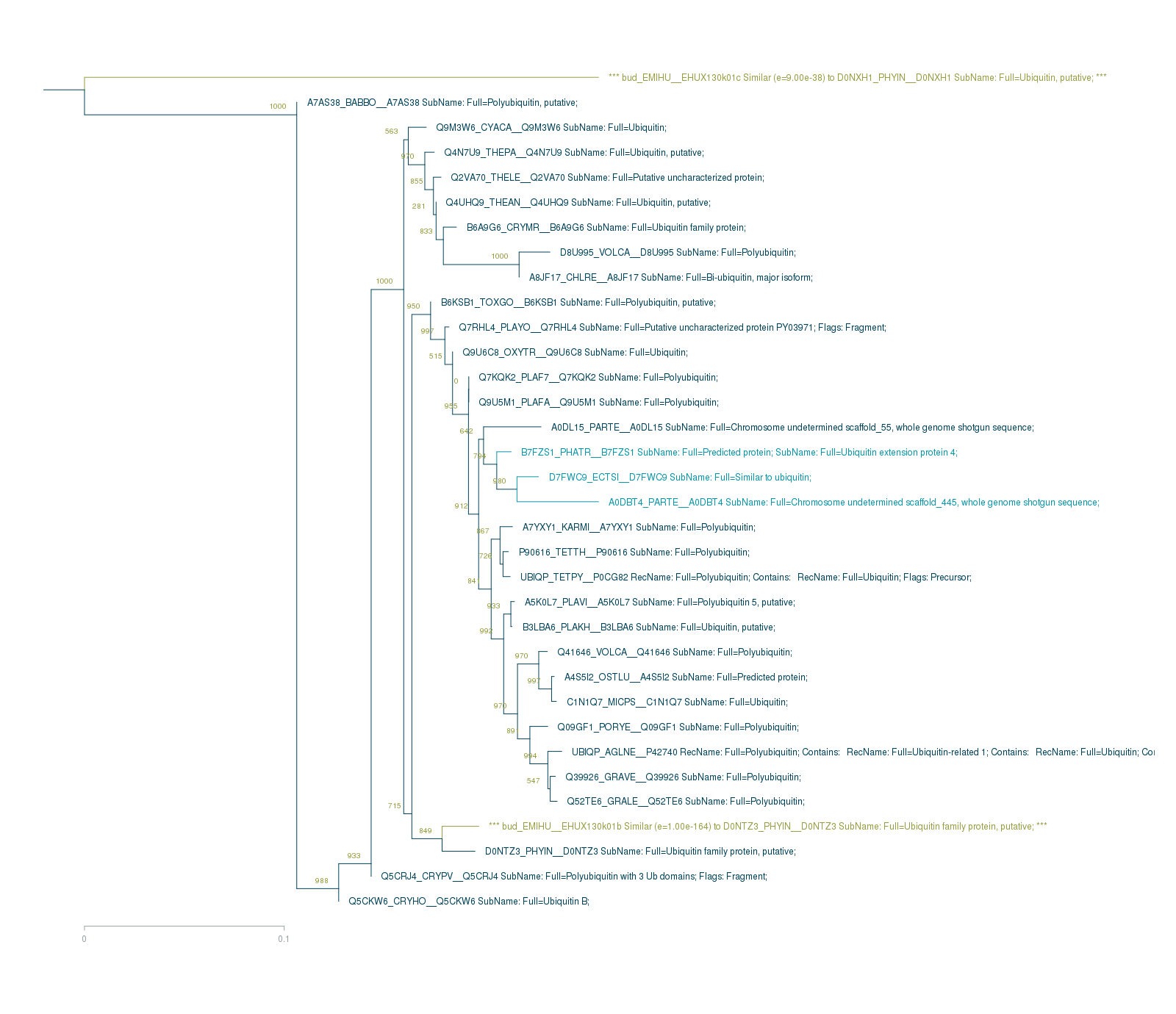

Supplement: Data S1 — Output files from BUDAPEST analysis. (ZIP) [file pone.0061868.s001.zip › BUDAPEST data/Jones_et_al_2012_Data/EHUX130k01c.png]

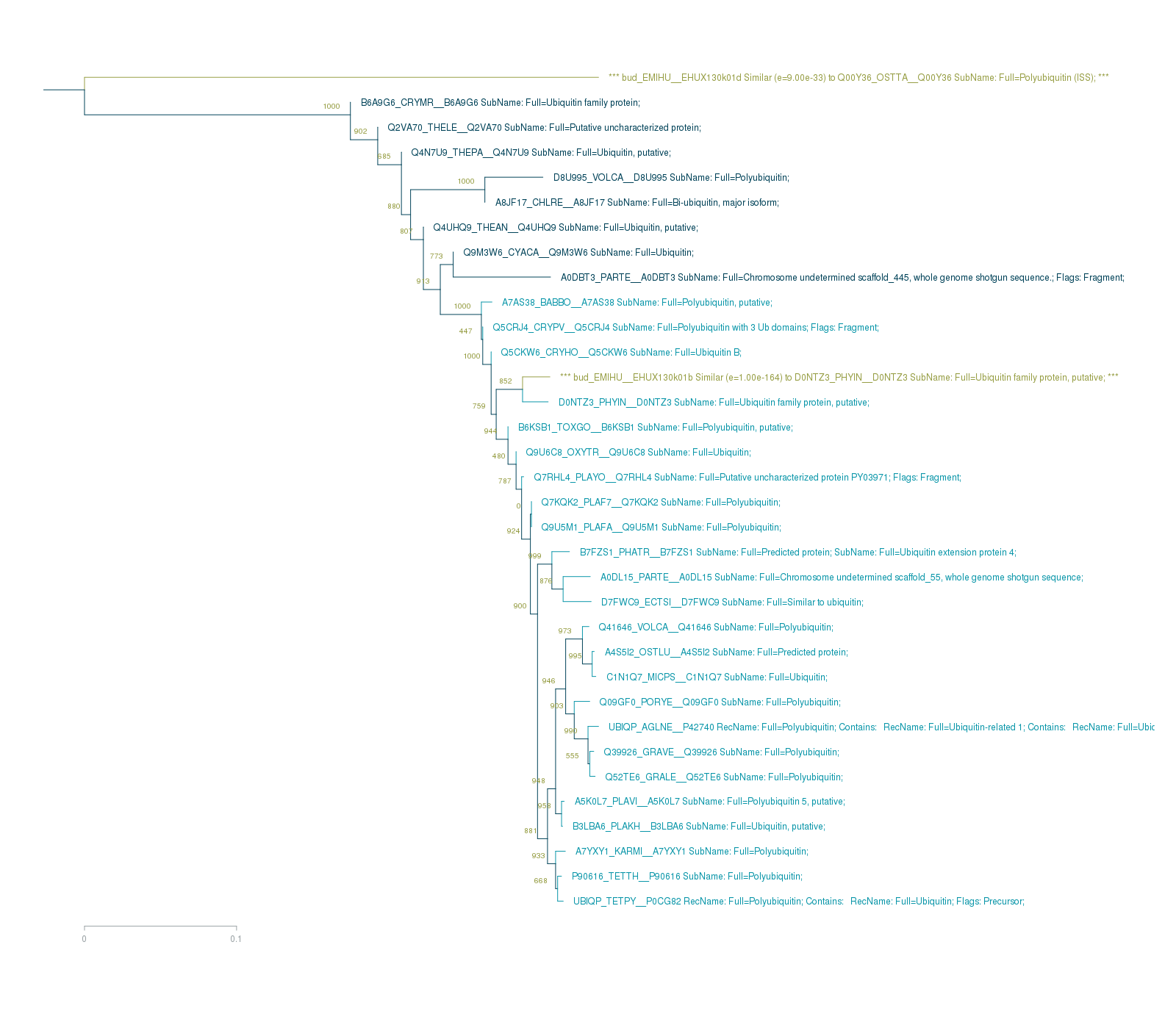

Supplement: Data S1 — Output files from BUDAPEST analysis. (ZIP) [file pone.0061868.s001.zip › BUDAPEST data/Jones_et_al_2012_Data/EHUX130k01d.png]

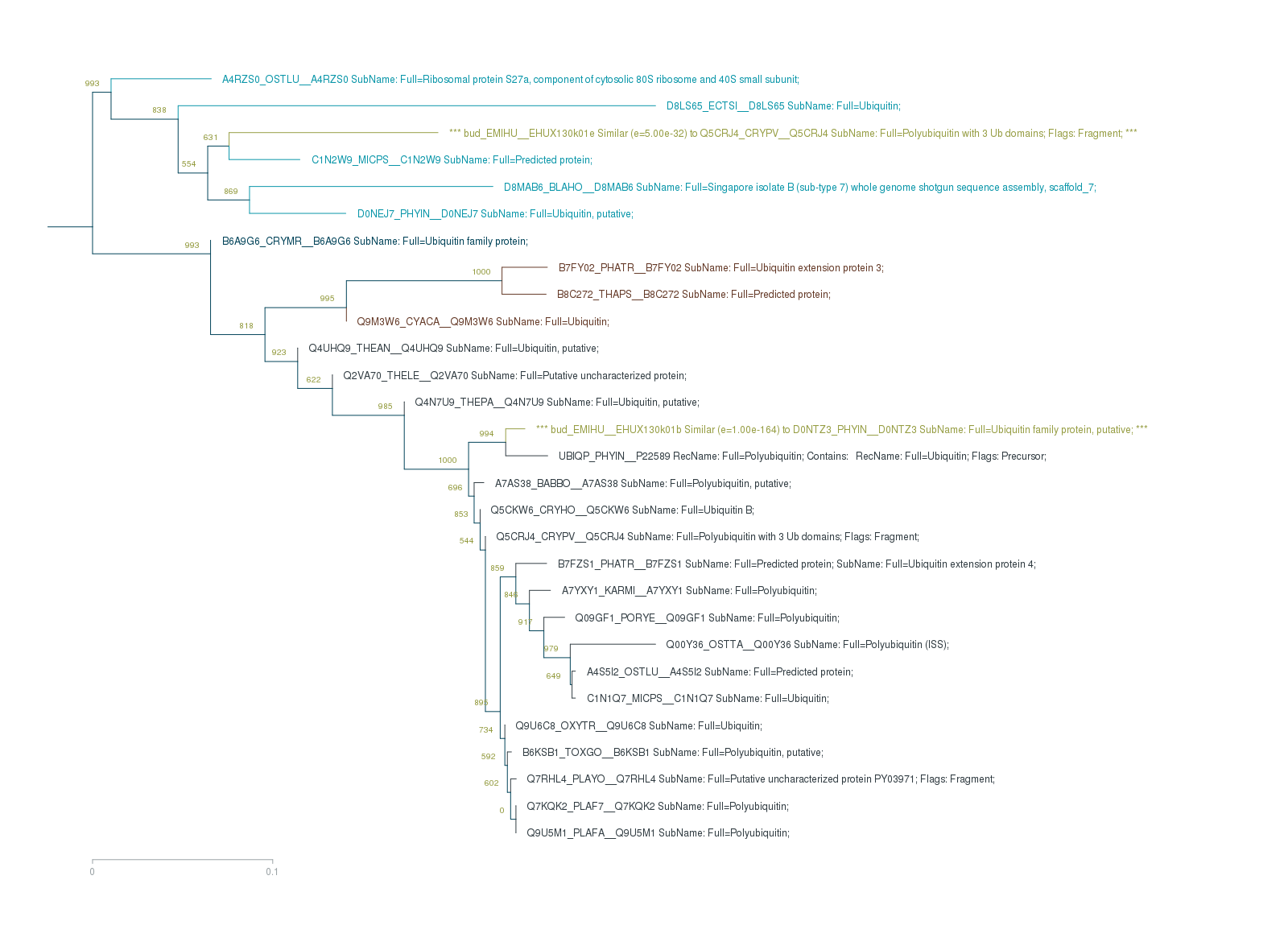

Supplement: Data S1 — Output files from BUDAPEST analysis. (ZIP) [file pone.0061868.s001.zip › BUDAPEST data/Jones_et_al_2012_Data/EHUX130k01e.png]

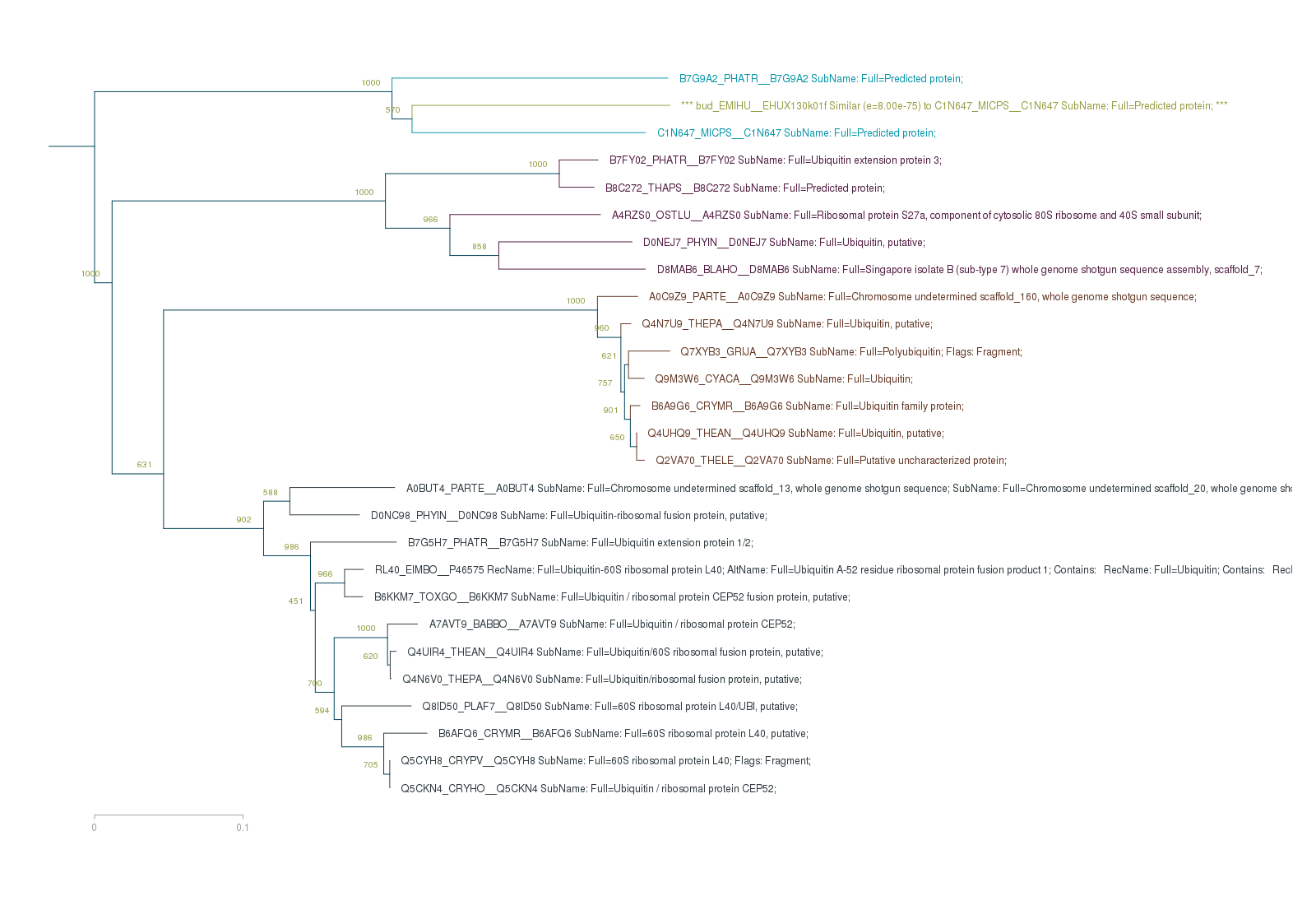

Supplement: Data S1 — Output files from BUDAPEST analysis. (ZIP) [file pone.0061868.s001.zip › BUDAPEST data/Jones_et_al_2012_Data/EHUX130k01f.png]

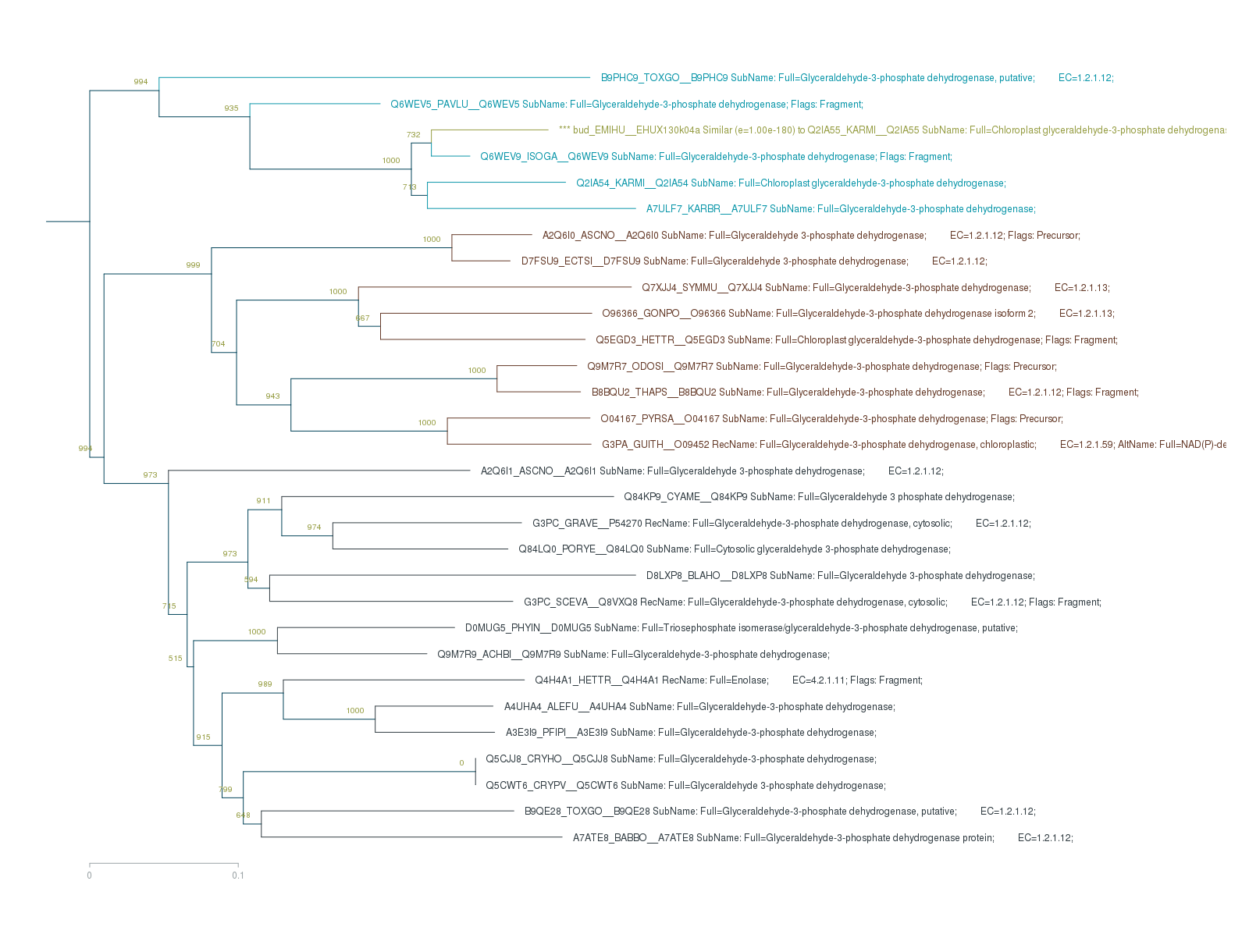

Supplement: Data S1 — Output files from BUDAPEST analysis. (ZIP) [file pone.0061868.s001.zip › BUDAPEST data/Jones_et_al_2012_Data/EHUX130k04a.png]

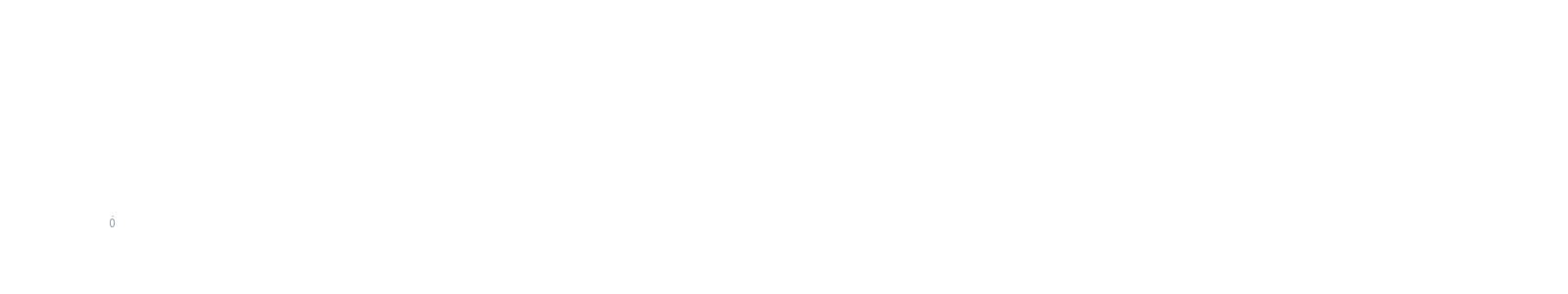

Supplement: Data S1 — Output files from BUDAPEST analysis. (ZIP) [file pone.0061868.s001.zip › BUDAPEST data/Jones_et_al_2012_Data/EHUX130k05a.png]

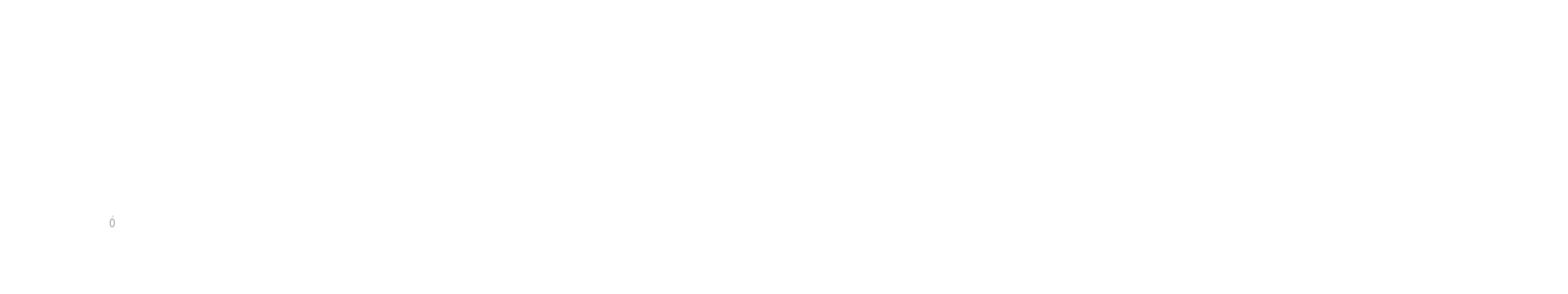

Supplement: Data S1 — Output files from BUDAPEST analysis. (ZIP) [file pone.0061868.s001.zip › BUDAPEST data/Jones_et_al_2012_Data/EHUX130k05b.png]

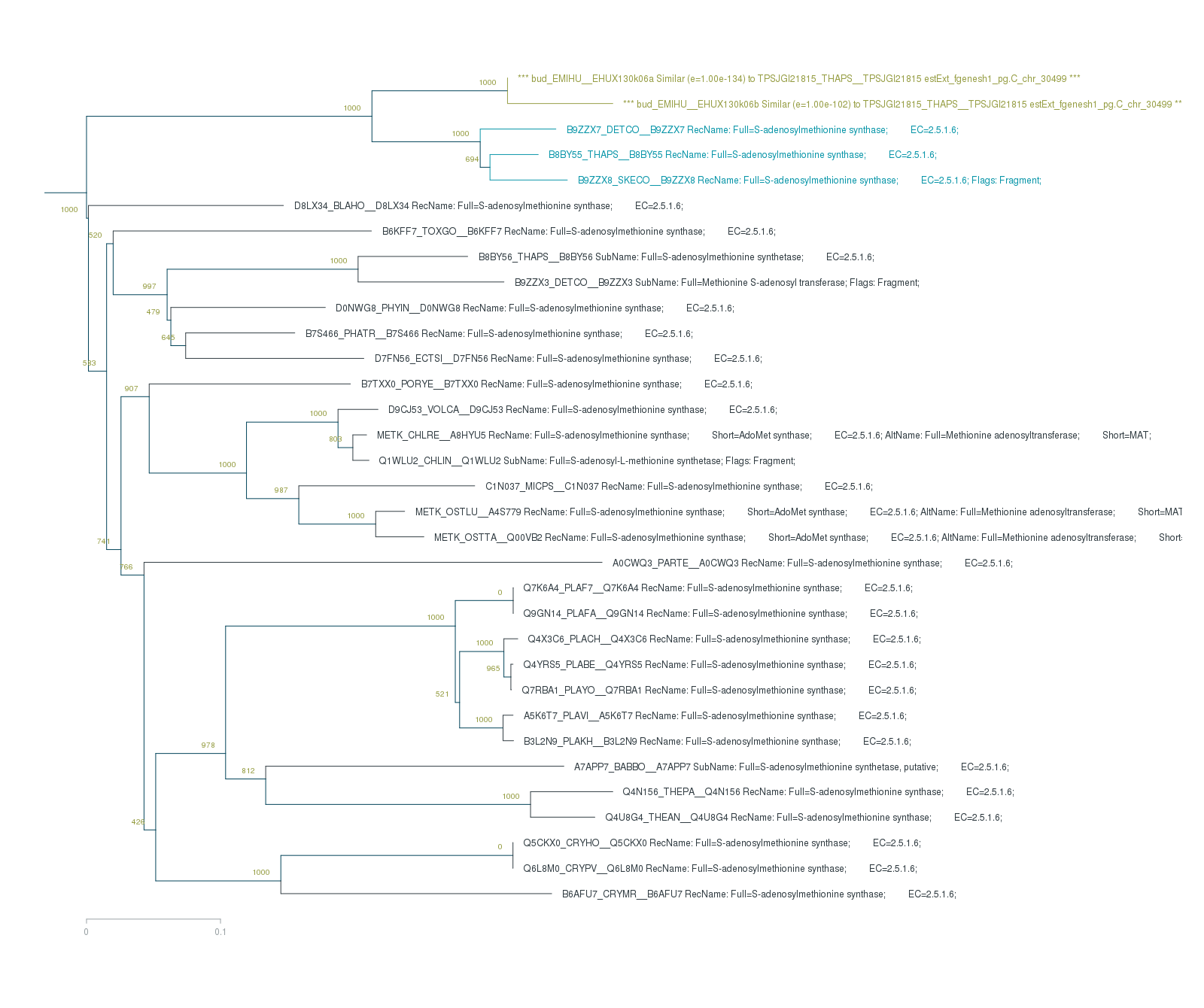

Supplement: Data S1 — Output files from BUDAPEST analysis. (ZIP) [file pone.0061868.s001.zip › BUDAPEST data/Jones_et_al_2012_Data/EHUX130k06a.png]

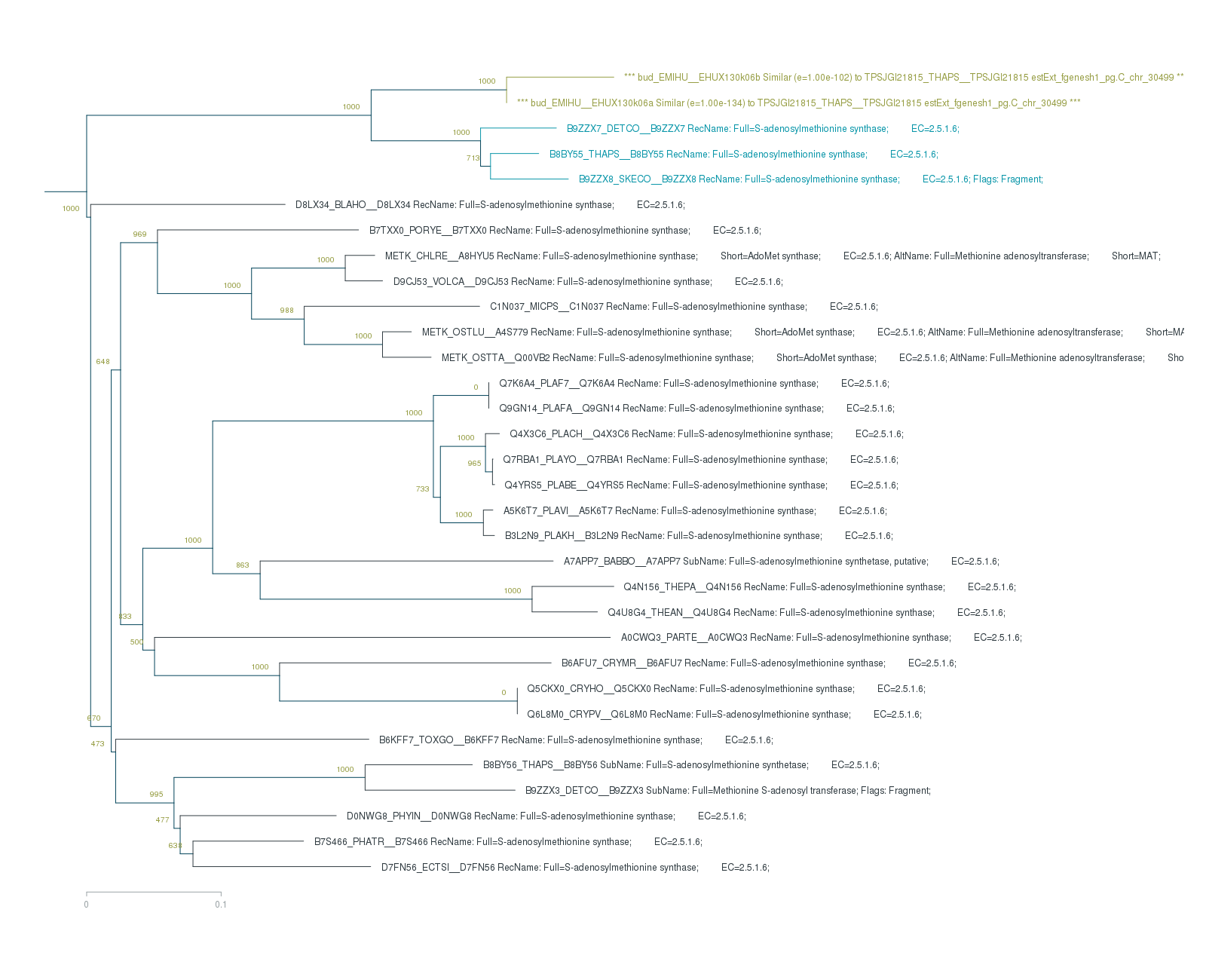

Supplement: Data S1 — Output files from BUDAPEST analysis. (ZIP) [file pone.0061868.s001.zip › BUDAPEST data/Jones_et_al_2012_Data/EHUX130k06b.png]

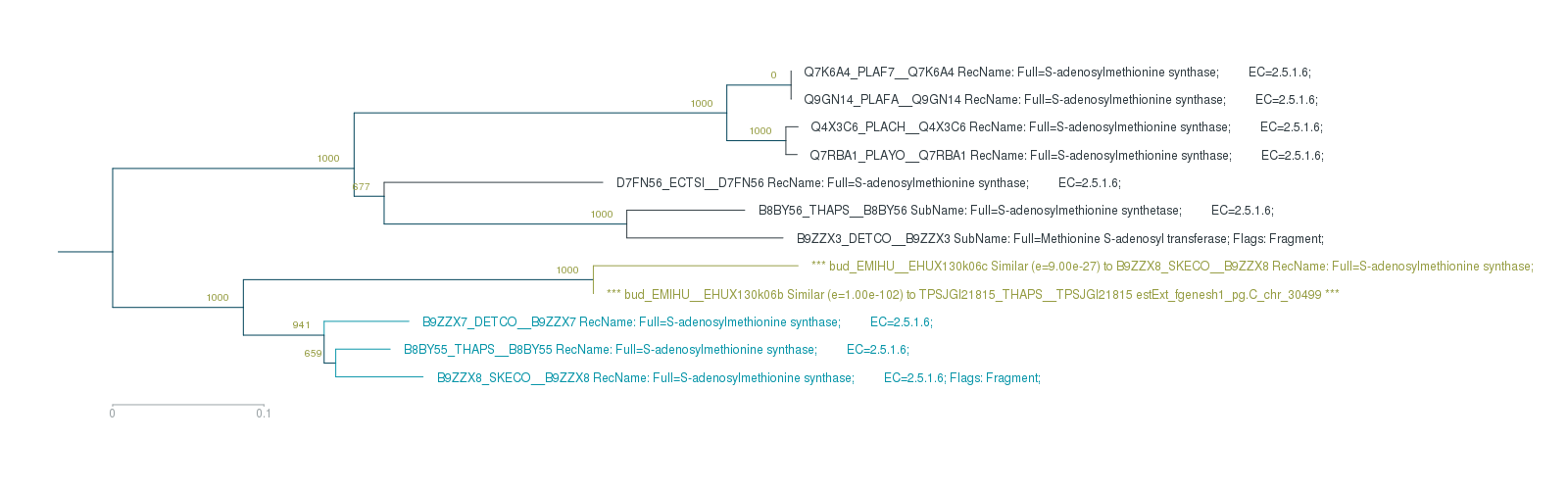

Supplement: Data S1 — Output files from BUDAPEST analysis. (ZIP) [file pone.0061868.s001.zip › BUDAPEST data/Jones_et_al_2012_Data/EHUX130k06c.png]

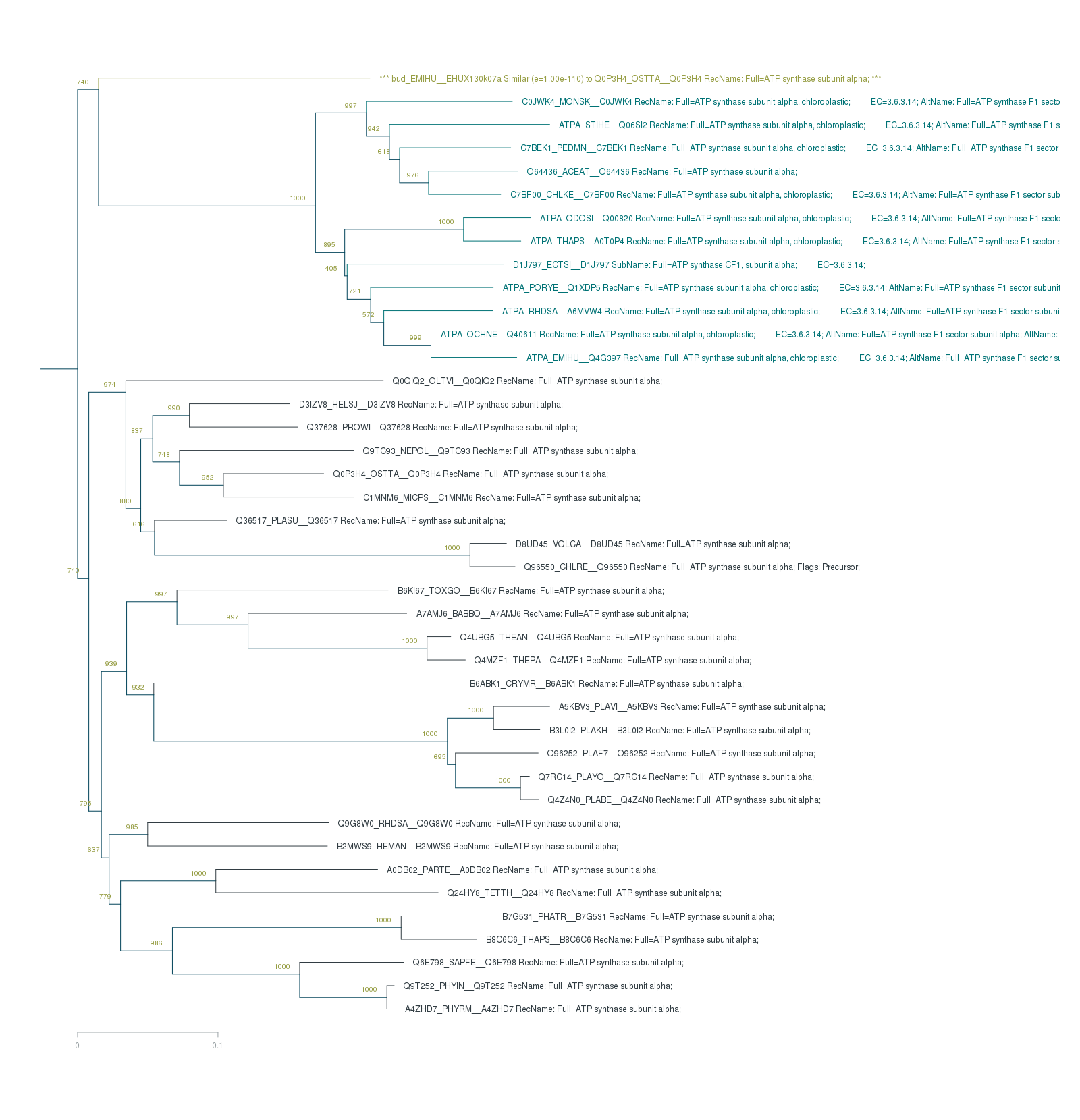

Supplement: Data S1 — Output files from BUDAPEST analysis. (ZIP) [file pone.0061868.s001.zip › BUDAPEST data/Jones_et_al_2012_Data/EHUX130k07a.png]

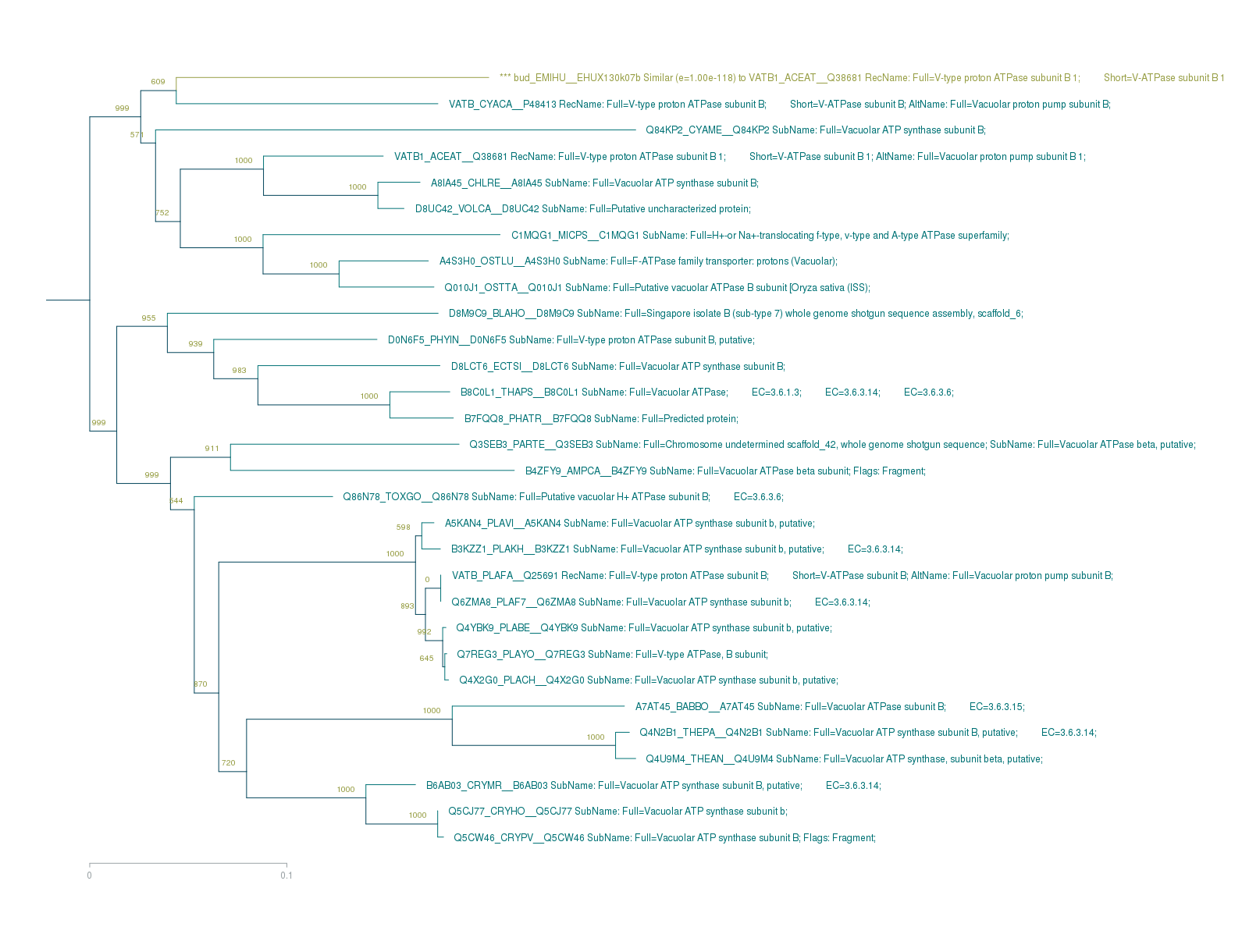

Supplement: Data S1 — Output files from BUDAPEST analysis. (ZIP) [file pone.0061868.s001.zip › BUDAPEST data/Jones_et_al_2012_Data/EHUX130k07b.png]

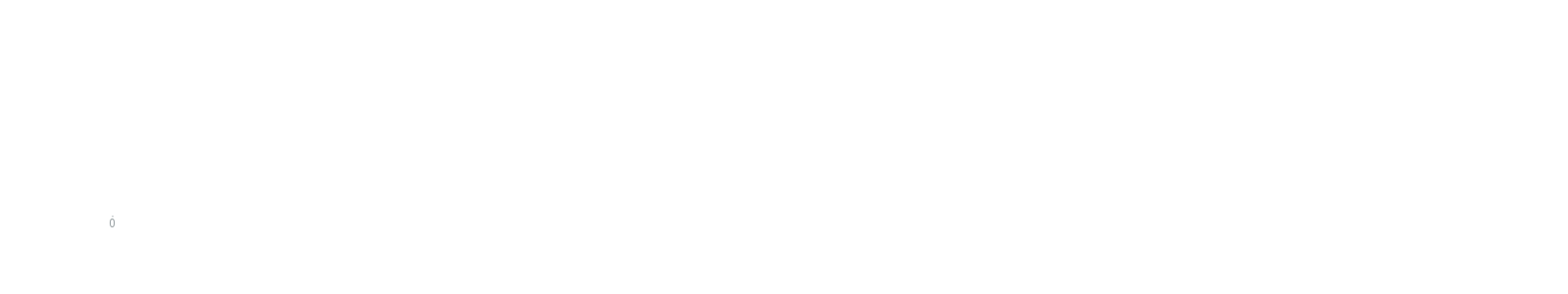

Supplement: Data S1 — Output files from BUDAPEST analysis. (ZIP) [file pone.0061868.s001.zip › BUDAPEST data/Jones_et_al_2012_Data/EHUX130k08a.png]

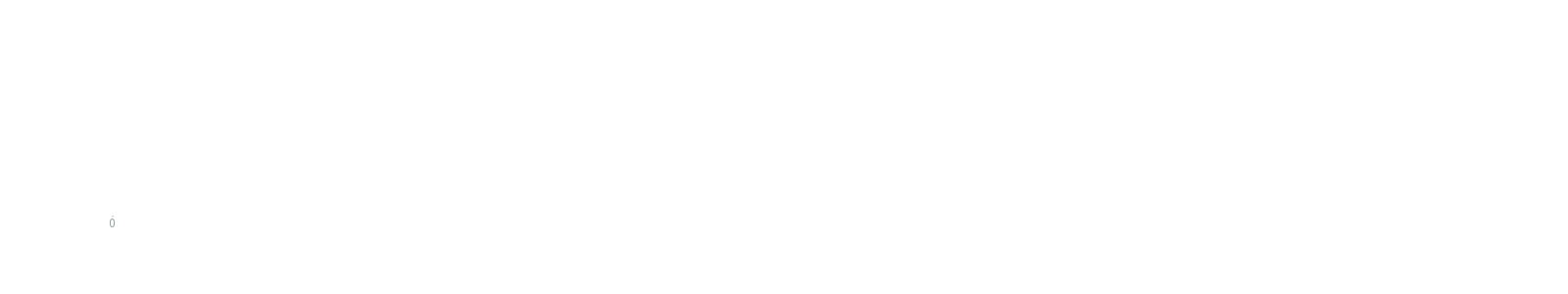

Supplement: Data S1 — Output files from BUDAPEST analysis. (ZIP) [file pone.0061868.s001.zip › BUDAPEST data/Jones_et_al_2012_Data/EHUX130k08b.png]

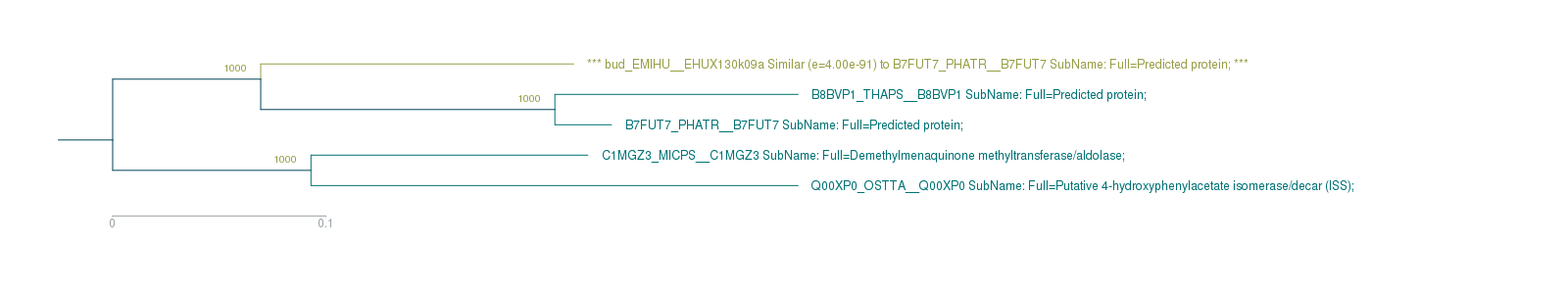

Supplement: Data S1 — Output files from BUDAPEST analysis. (ZIP) [file pone.0061868.s001.zip › BUDAPEST data/Jones_et_al_2012_Data/EHUX130k09a.png]

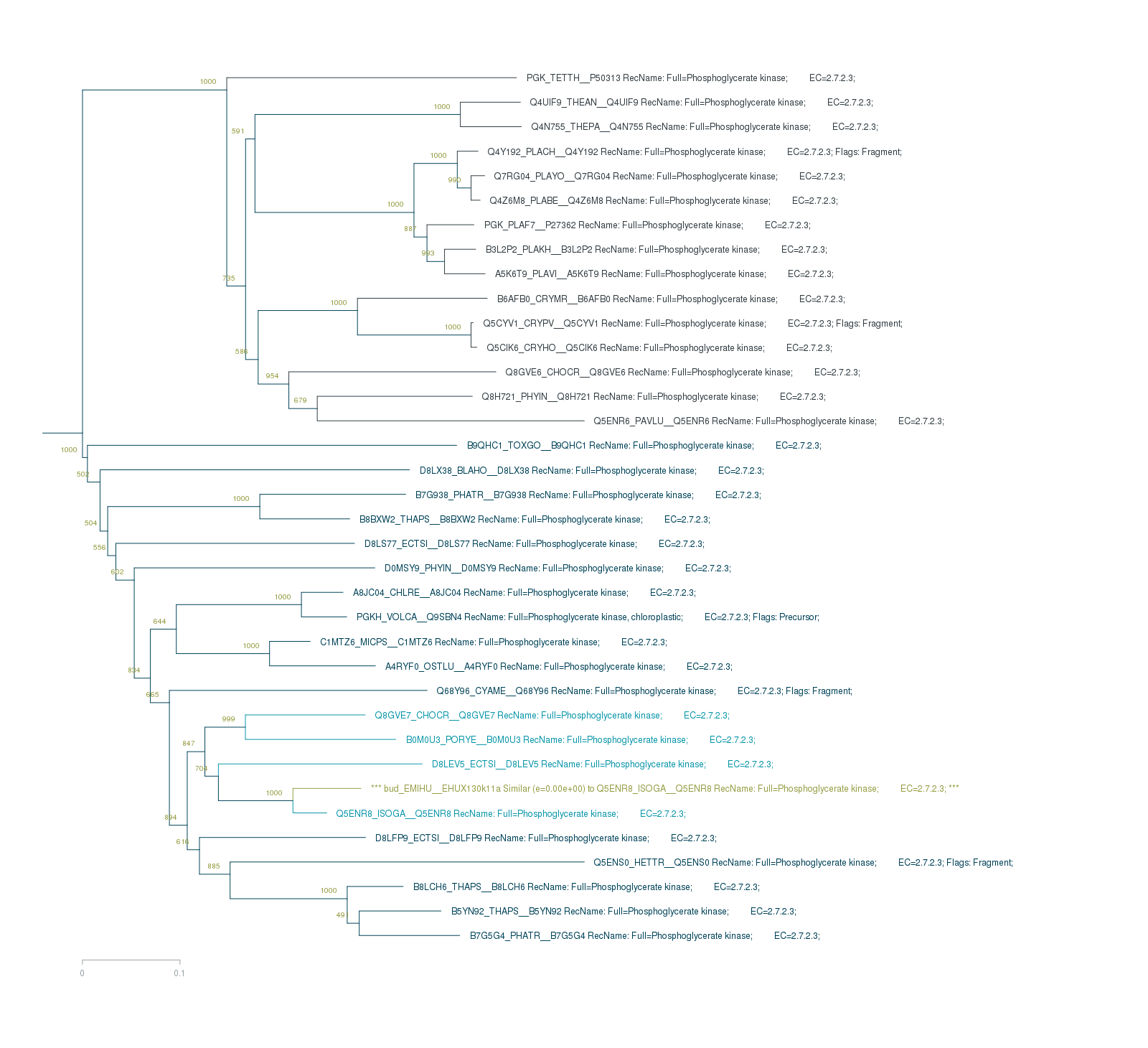

Supplement: Data S1 — Output files from BUDAPEST analysis. (ZIP) [file pone.0061868.s001.zip › BUDAPEST data/Jones_et_al_2012_Data/EHUX130k11a.png]

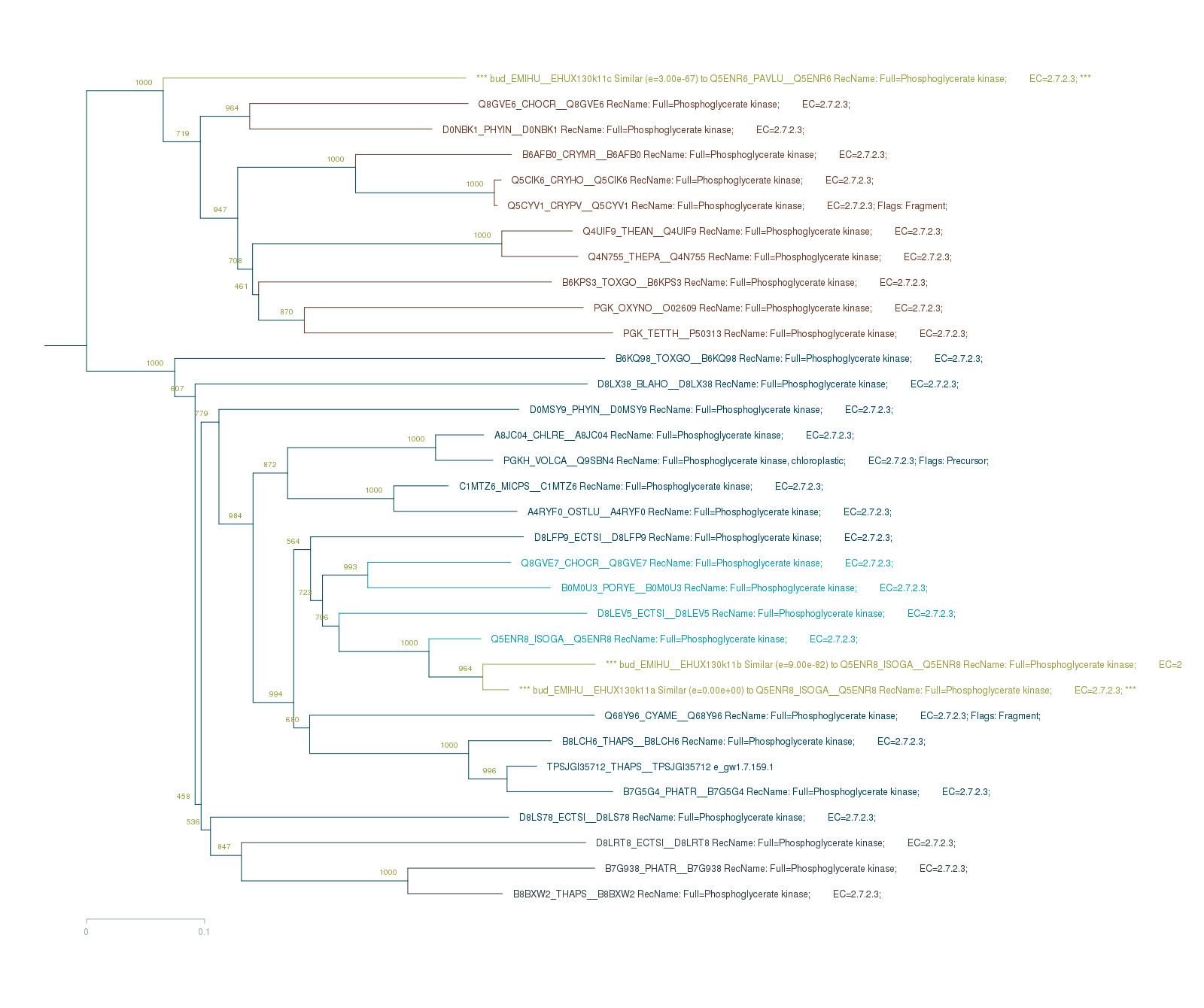

Supplement: Data S1 — Output files from BUDAPEST analysis. (ZIP) [file pone.0061868.s001.zip › BUDAPEST data/Jones_et_al_2012_Data/EHUX130k11b.png]

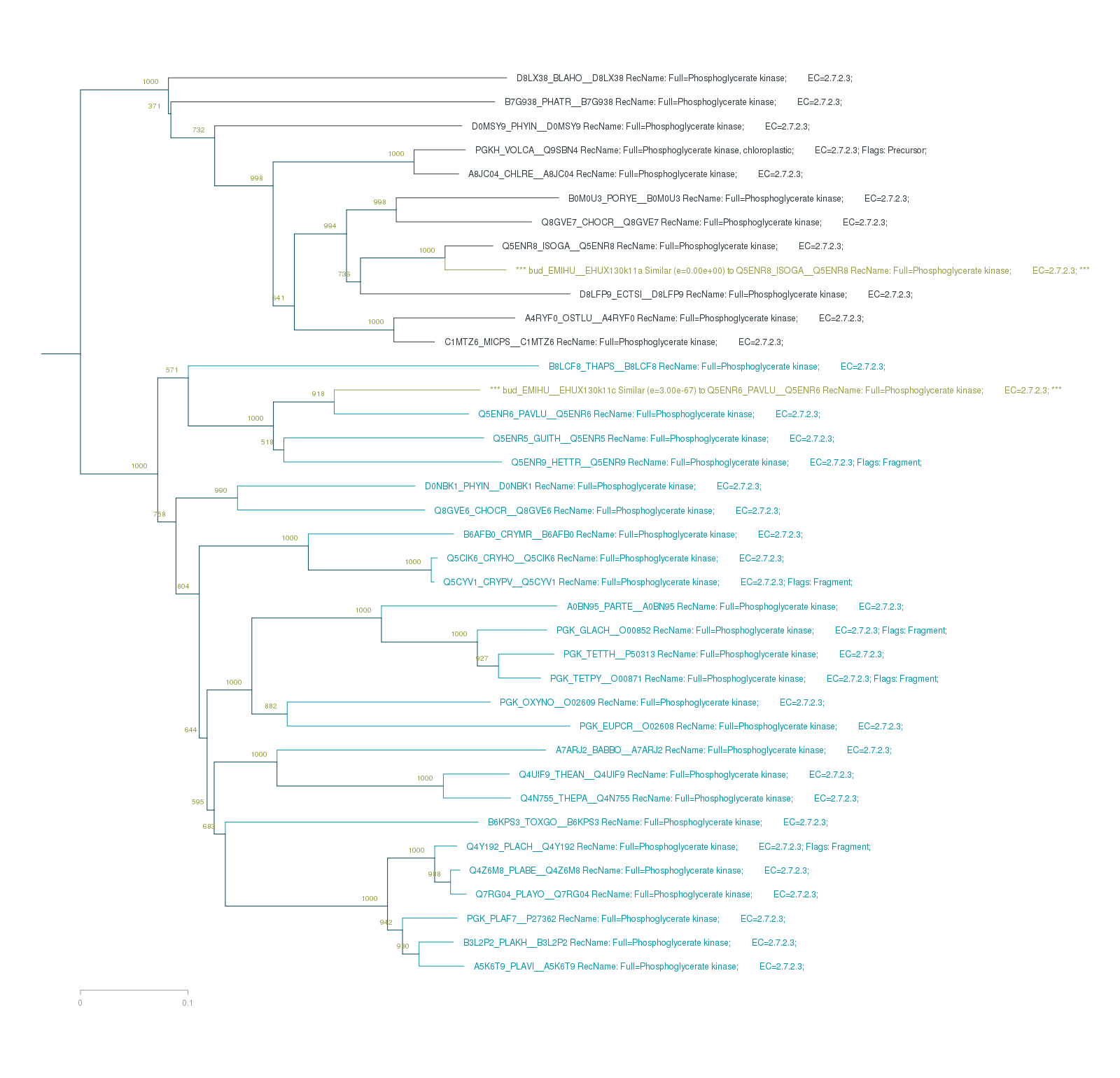

Supplement: Data S1 — Output files from BUDAPEST analysis. (ZIP) [file pone.0061868.s001.zip › BUDAPEST data/Jones_et_al_2012_Data/EHUX130k11c.png]

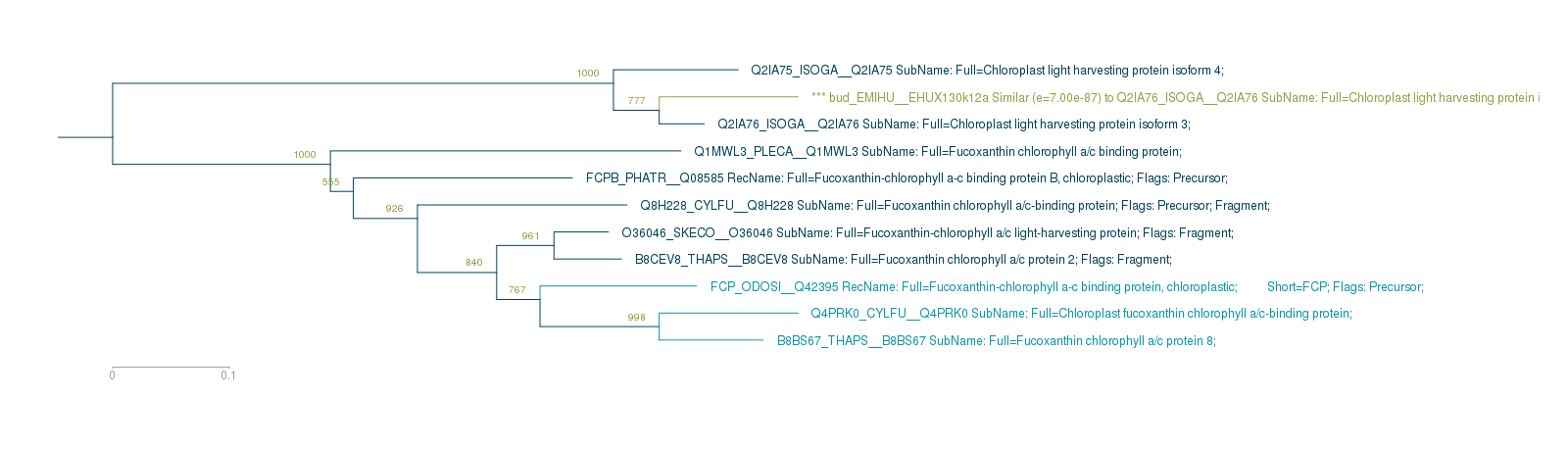

Supplement: Data S1 — Output files from BUDAPEST analysis. (ZIP) [file pone.0061868.s001.zip › BUDAPEST data/Jones_et_al_2012_Data/EHUX130k12a.png]

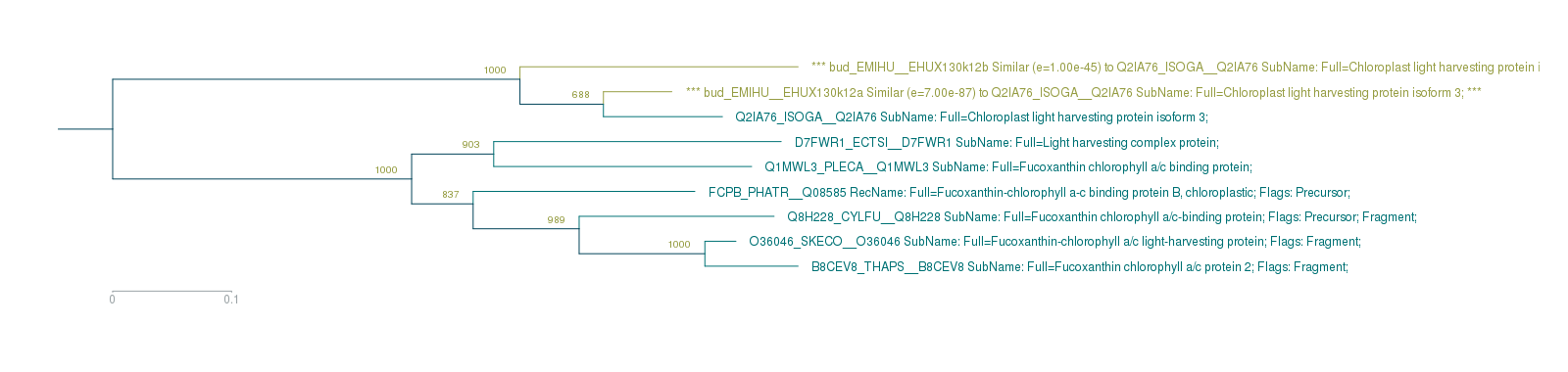

Supplement: Data S1 — Output files from BUDAPEST analysis. (ZIP) [file pone.0061868.s001.zip › BUDAPEST data/Jones_et_al_2012_Data/EHUX130k12b.png]

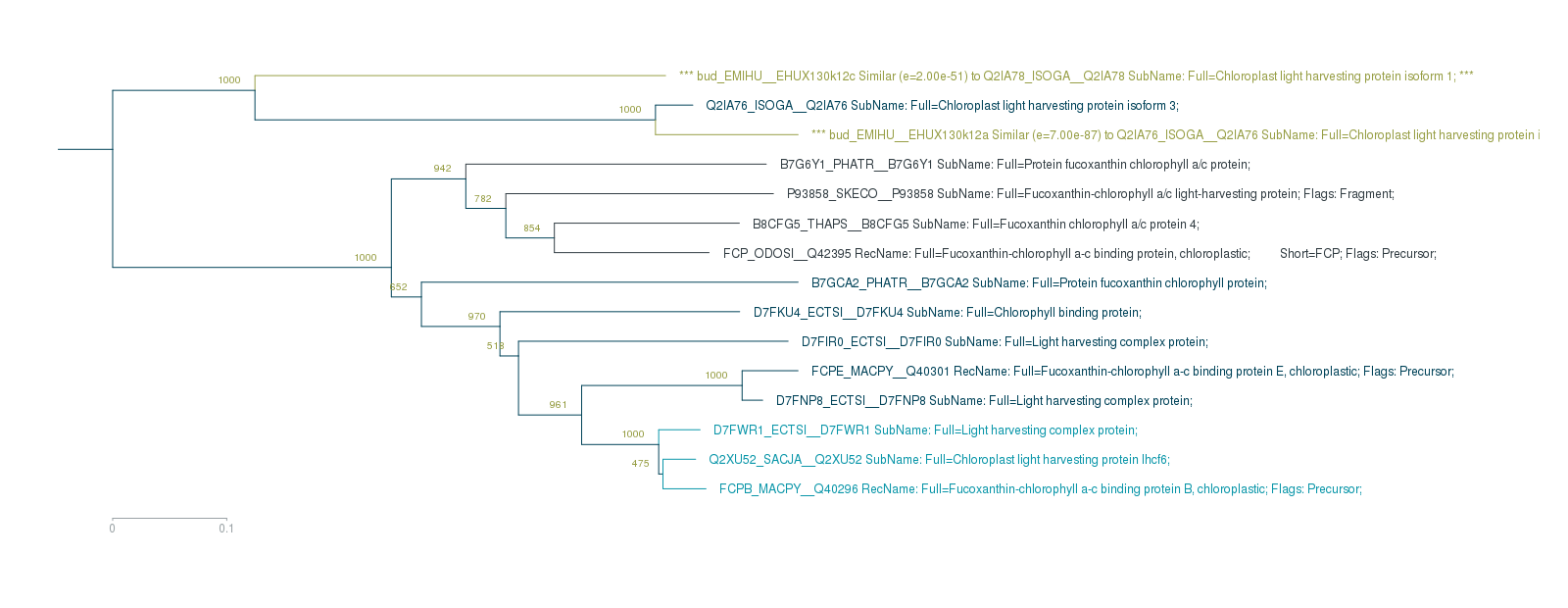

Supplement: Data S1 — Output files from BUDAPEST analysis. (ZIP) [file pone.0061868.s001.zip › BUDAPEST data/Jones_et_al_2012_Data/EHUX130k12c.png]

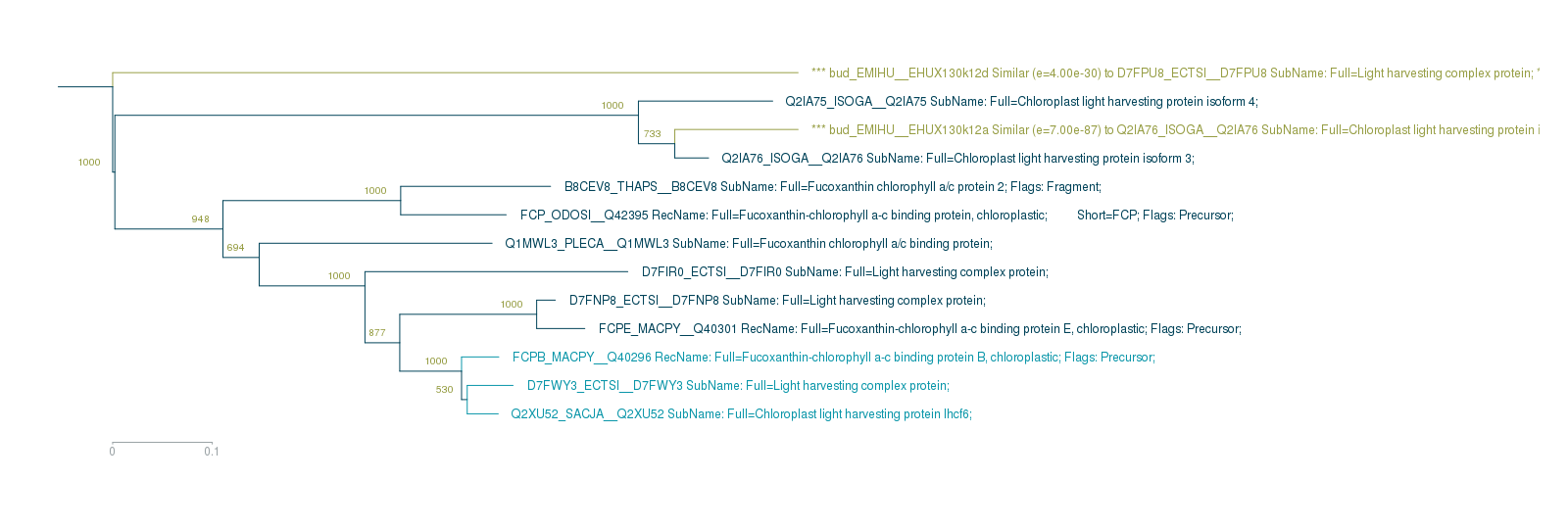

Supplement: Data S1 — Output files from BUDAPEST analysis. (ZIP) [file pone.0061868.s001.zip › BUDAPEST data/Jones_et_al_2012_Data/EHUX130k12d.png]

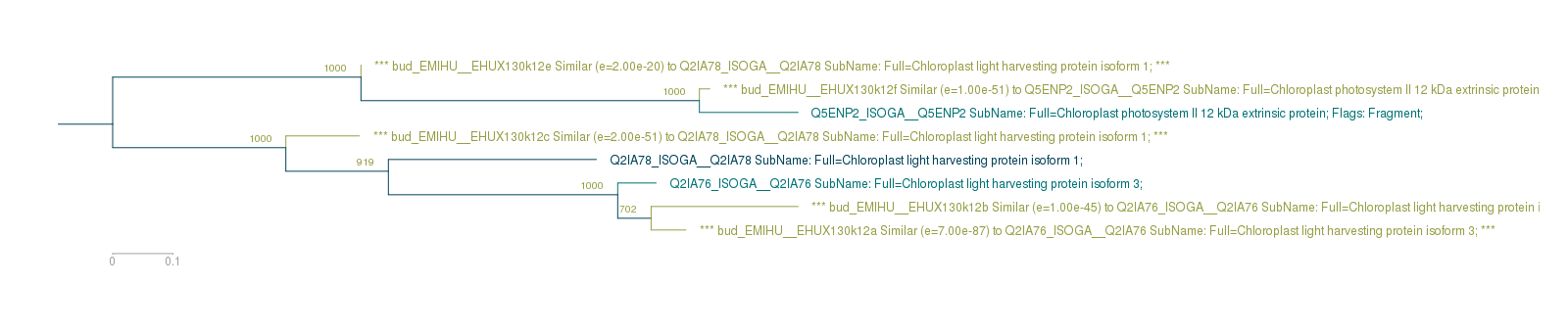

Supplement: Data S1 — Output files from BUDAPEST analysis. (ZIP) [file pone.0061868.s001.zip › BUDAPEST data/Jones_et_al_2012_Data/EHUX130k12e.png]

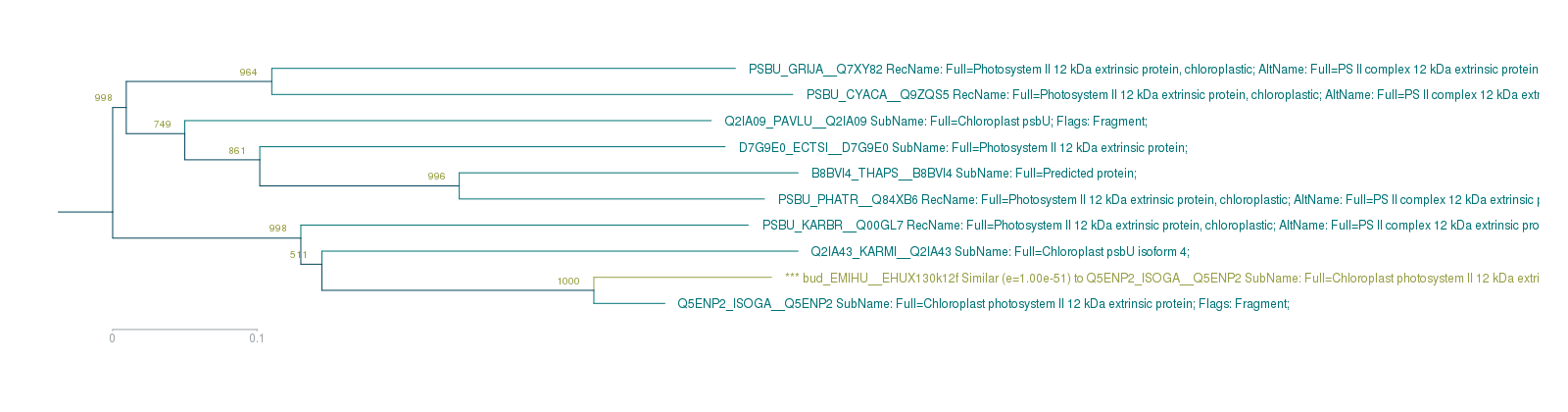

Supplement: Data S1 — Output files from BUDAPEST analysis. (ZIP) [file pone.0061868.s001.zip › BUDAPEST data/Jones_et_al_2012_Data/EHUX130k12f.png]

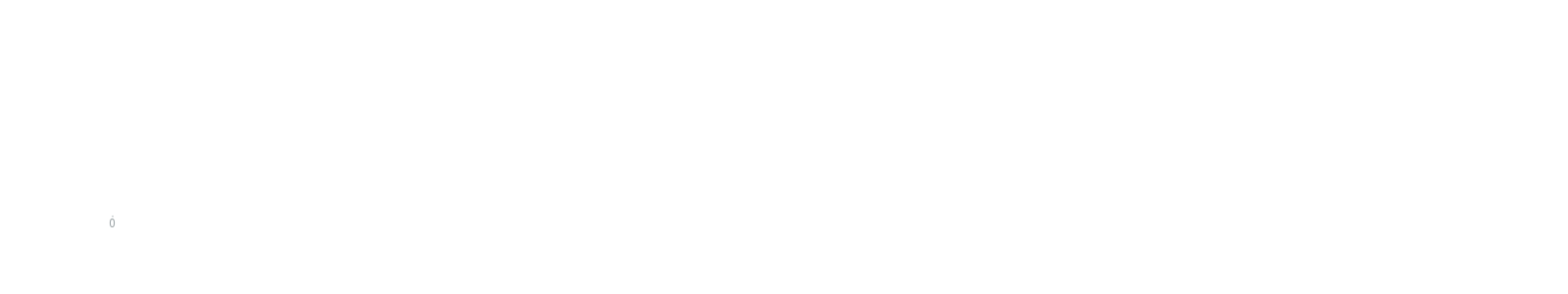

Supplement: Data S1 — Output files from BUDAPEST analysis. (ZIP) [file pone.0061868.s001.zip › BUDAPEST data/Jones_et_al_2012_Data/EHUX130k13a.png]

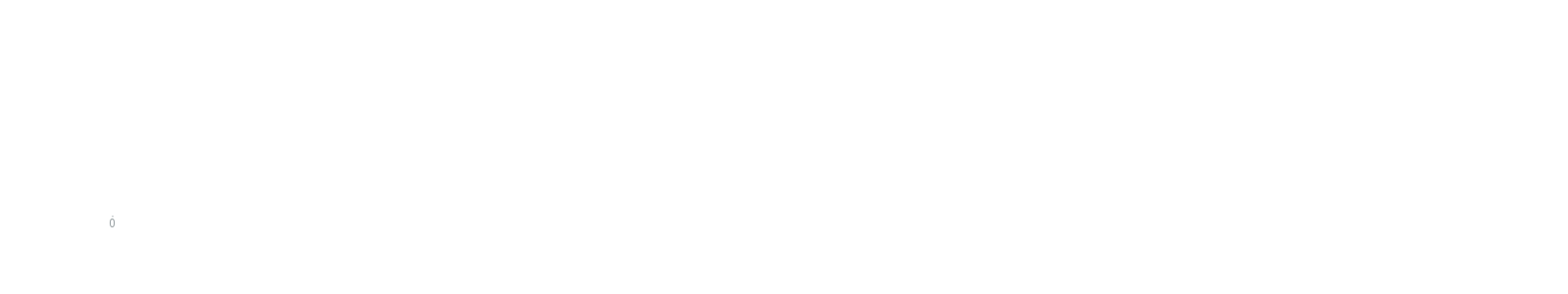

Supplement: Data S1 — Output files from BUDAPEST analysis. (ZIP) [file pone.0061868.s001.zip › BUDAPEST data/Jones_et_al_2012_Data/EHUX130k13b.png]

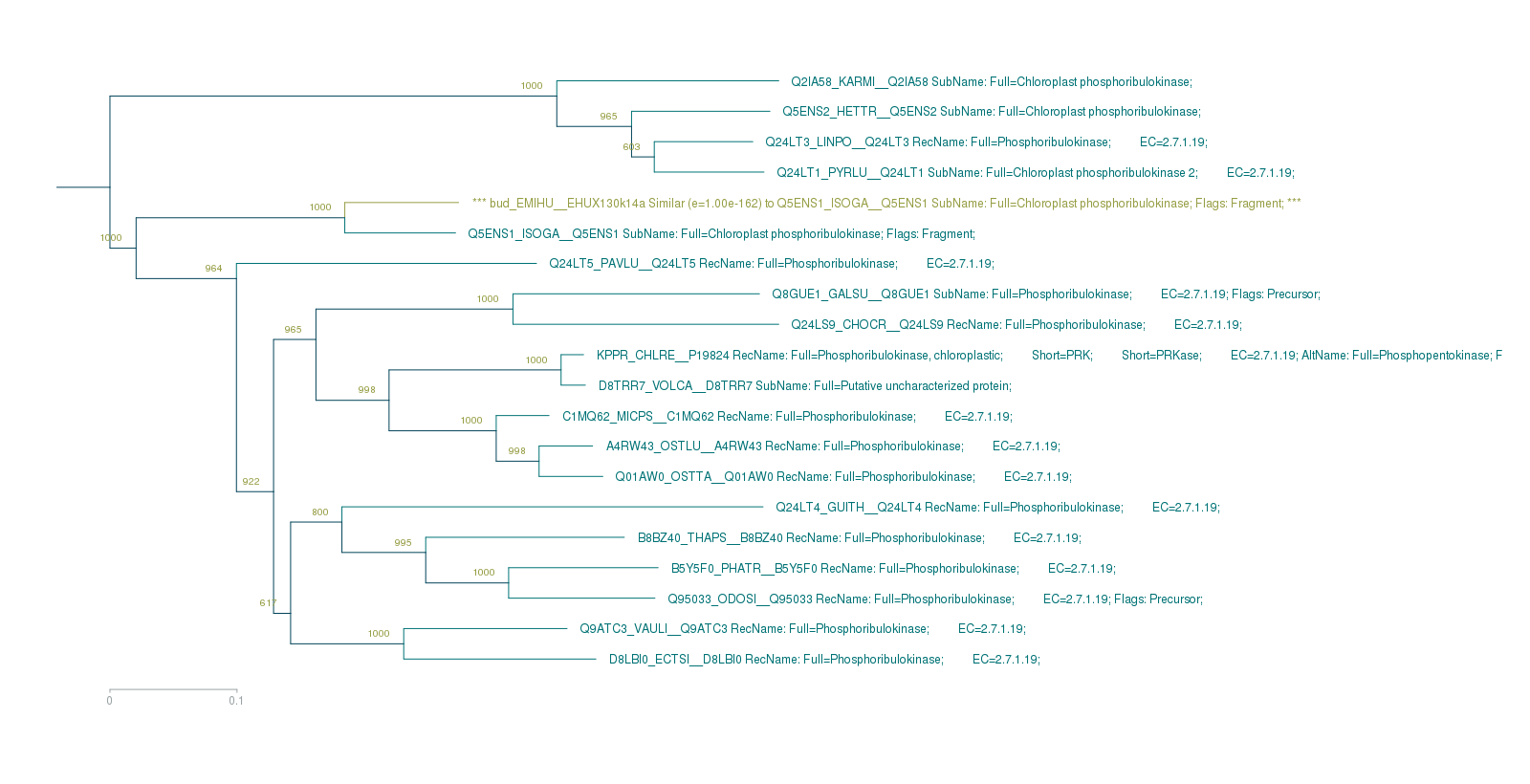

Supplement: Data S1 — Output files from BUDAPEST analysis. (ZIP) [file pone.0061868.s001.zip › BUDAPEST data/Jones_et_al_2012_Data/EHUX130k14a.png]

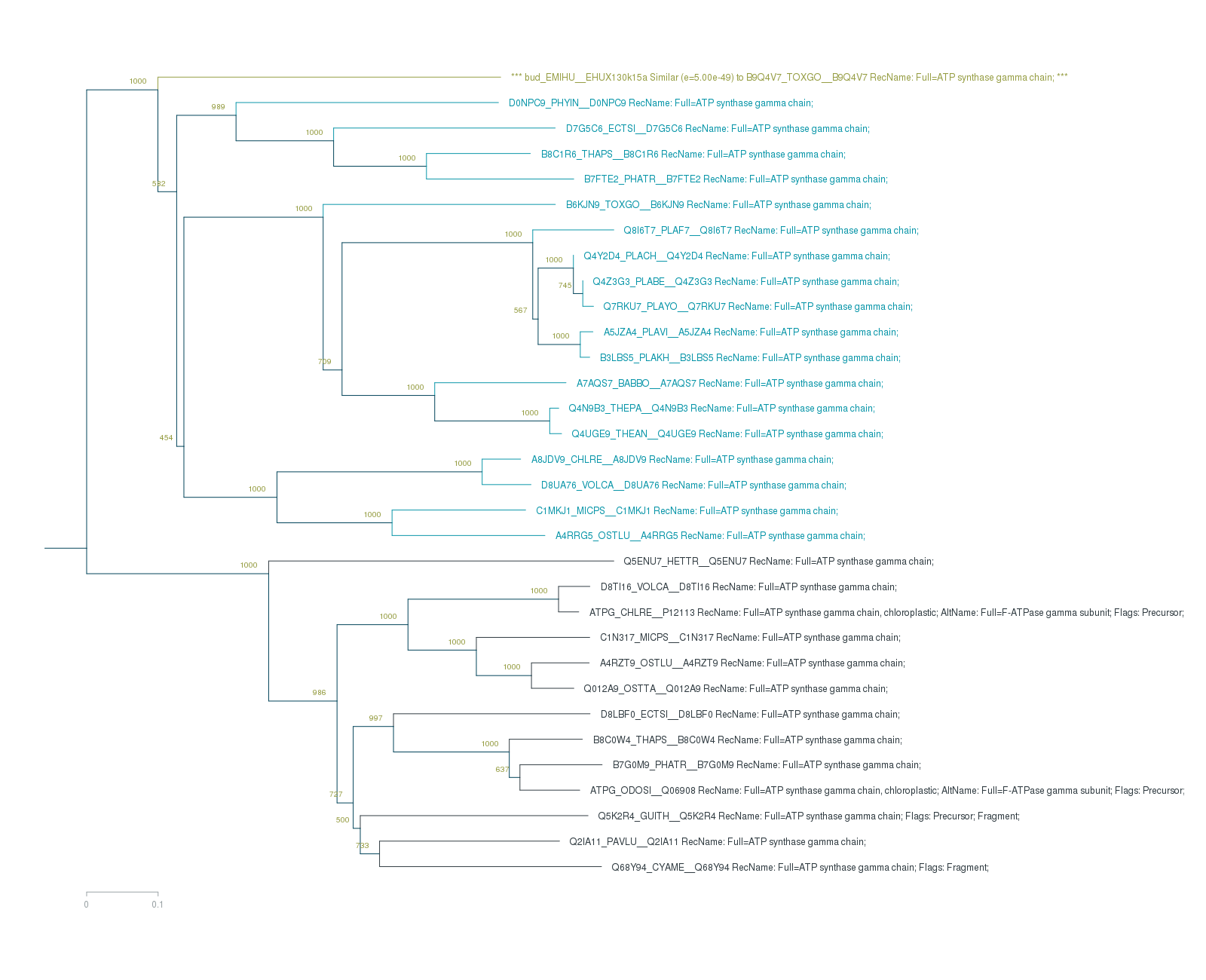

Supplement: Data S1 — Output files from BUDAPEST analysis. (ZIP) [file pone.0061868.s001.zip › BUDAPEST data/Jones_et_al_2012_Data/EHUX130k15a.png]

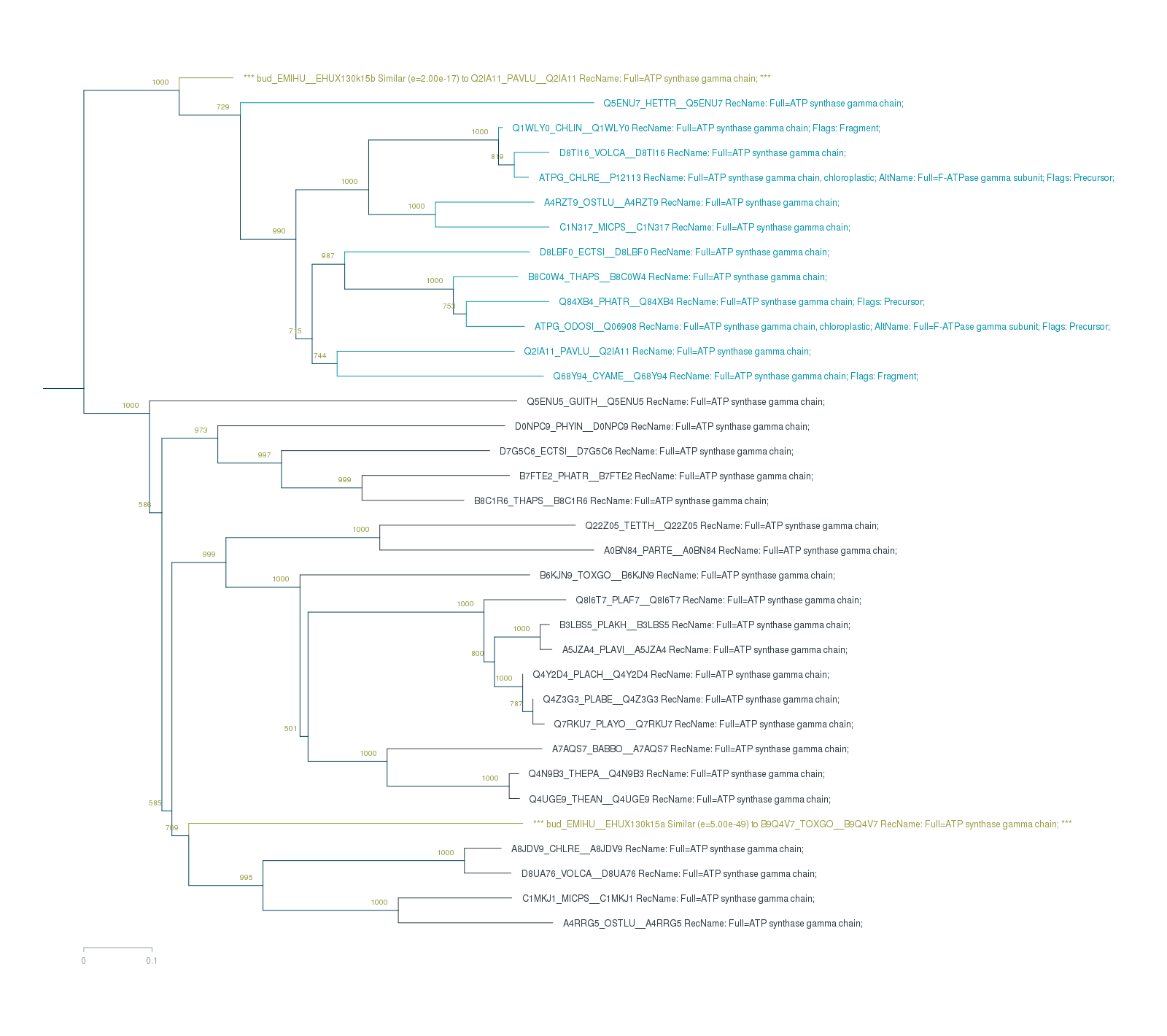

Supplement: Data S1 — Output files from BUDAPEST analysis. (ZIP) [file pone.0061868.s001.zip › BUDAPEST data/Jones_et_al_2012_Data/EHUX130k15b.png]

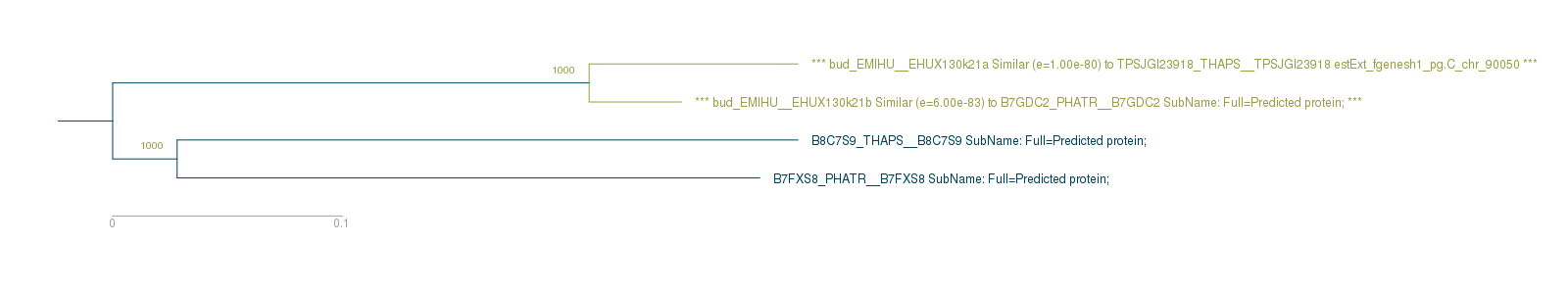

Supplement: Data S1 — Output files from BUDAPEST analysis. (ZIP) [file pone.0061868.s001.zip › BUDAPEST data/Jones_et_al_2012_Data/EHUX130k21a.png]

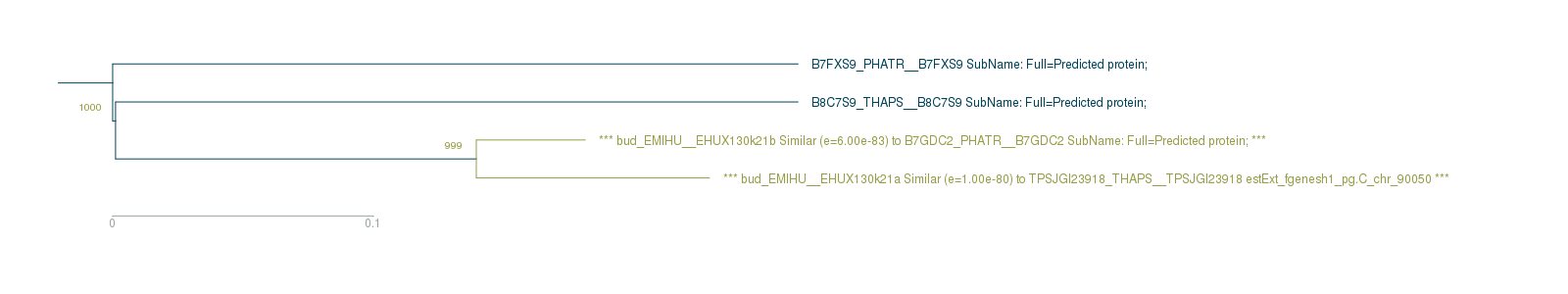

Supplement: Data S1 — Output files from BUDAPEST analysis. (ZIP) [file pone.0061868.s001.zip › BUDAPEST data/Jones_et_al_2012_Data/EHUX130k21b.png]

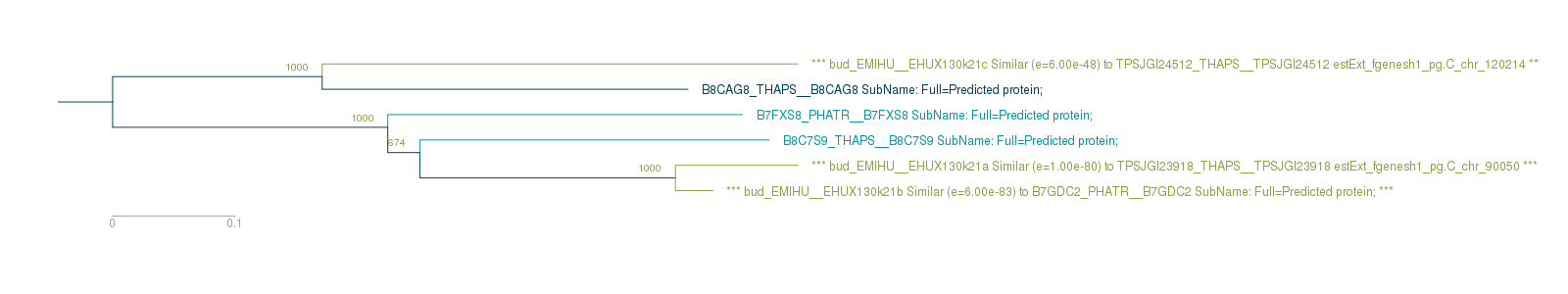

Supplement: Data S1 — Output files from BUDAPEST analysis. (ZIP) [file pone.0061868.s001.zip › BUDAPEST data/Jones_et_al_2012_Data/EHUX130k21c.png]

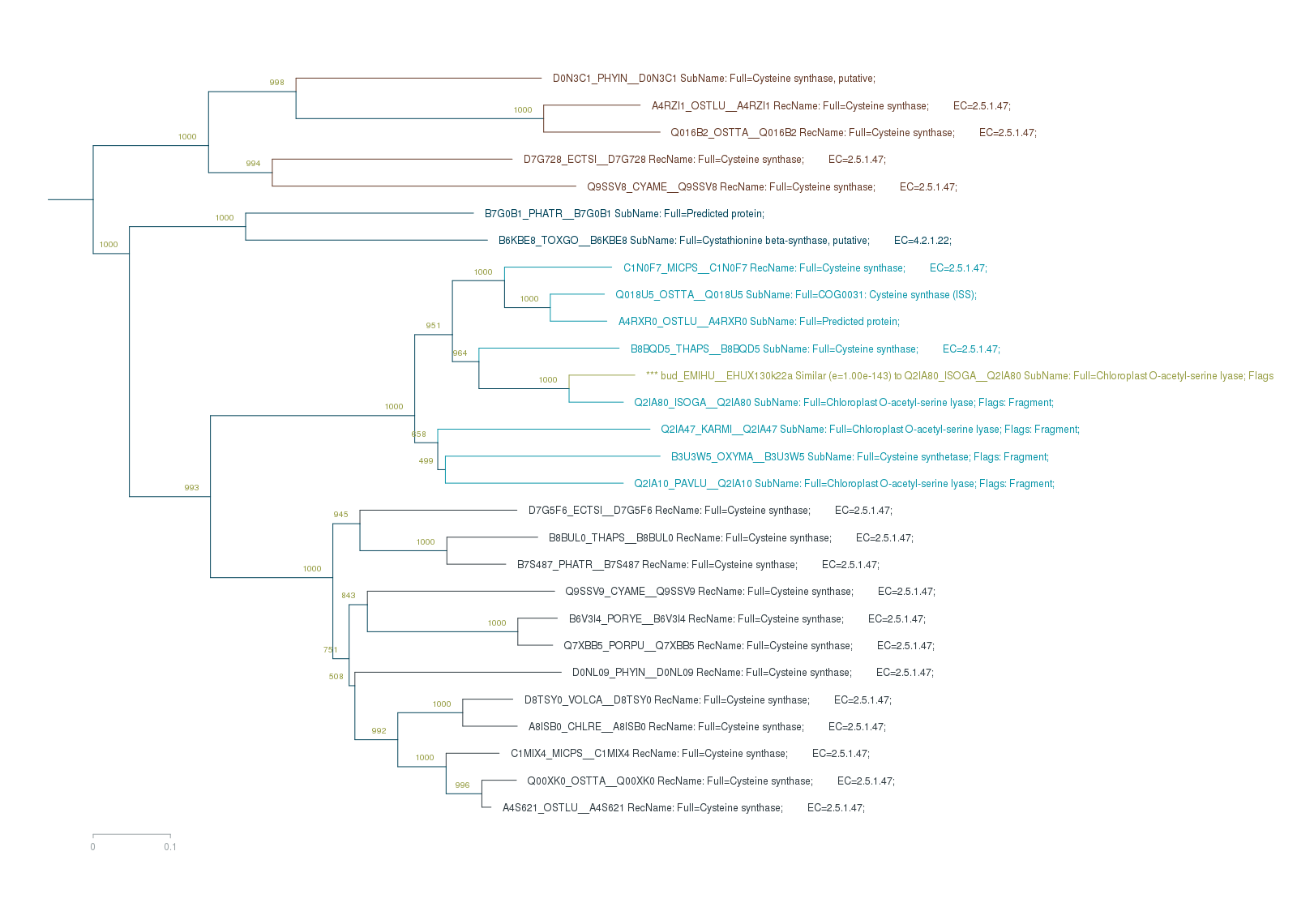

Supplement: Data S1 — Output files from BUDAPEST analysis. (ZIP) [file pone.0061868.s001.zip › BUDAPEST data/Jones_et_al_2012_Data/EHUX130k22a.png]

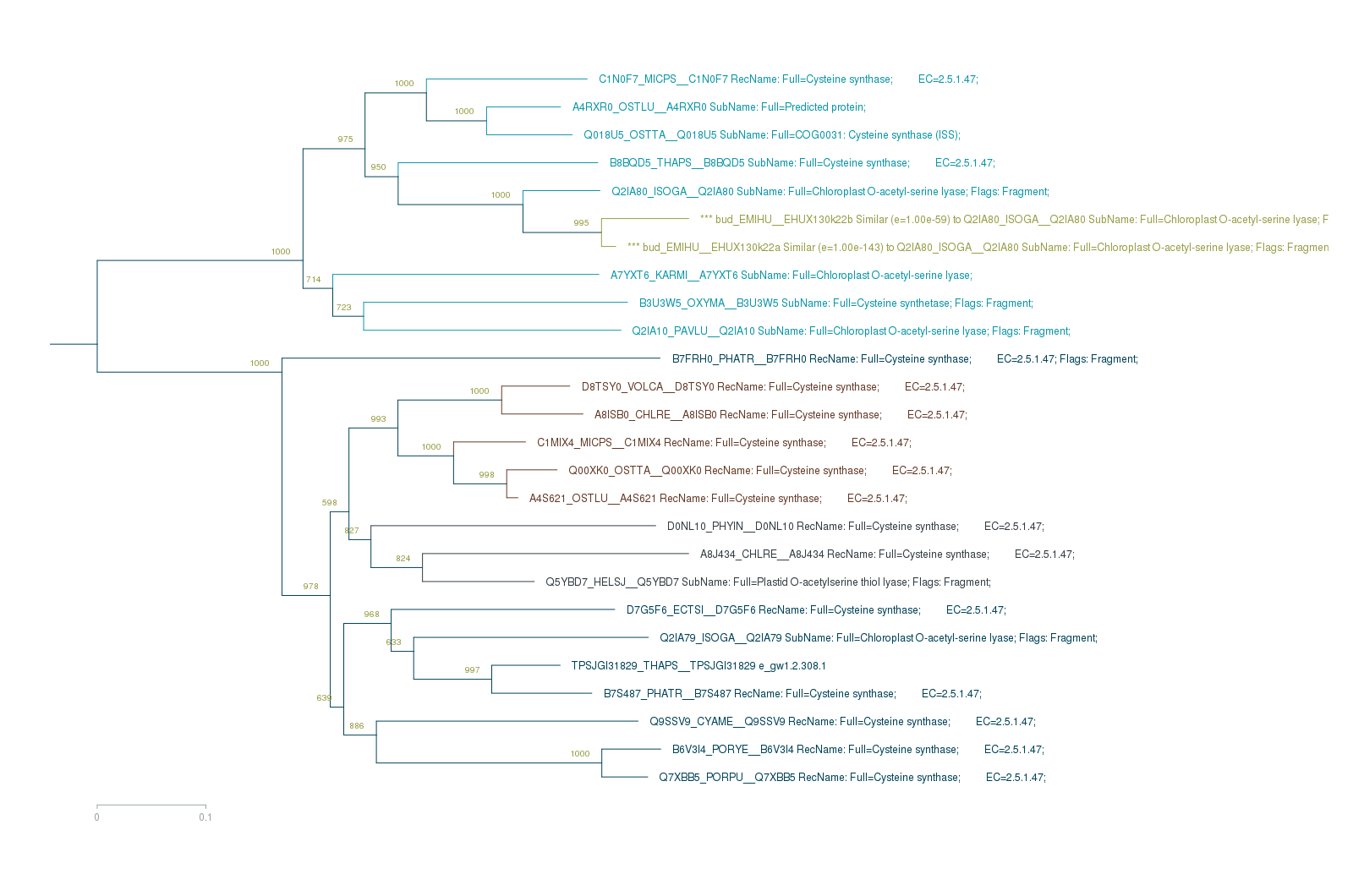

Supplement: Data S1 — Output files from BUDAPEST analysis. (ZIP) [file pone.0061868.s001.zip › BUDAPEST data/Jones_et_al_2012_Data/EHUX130k22b.png]

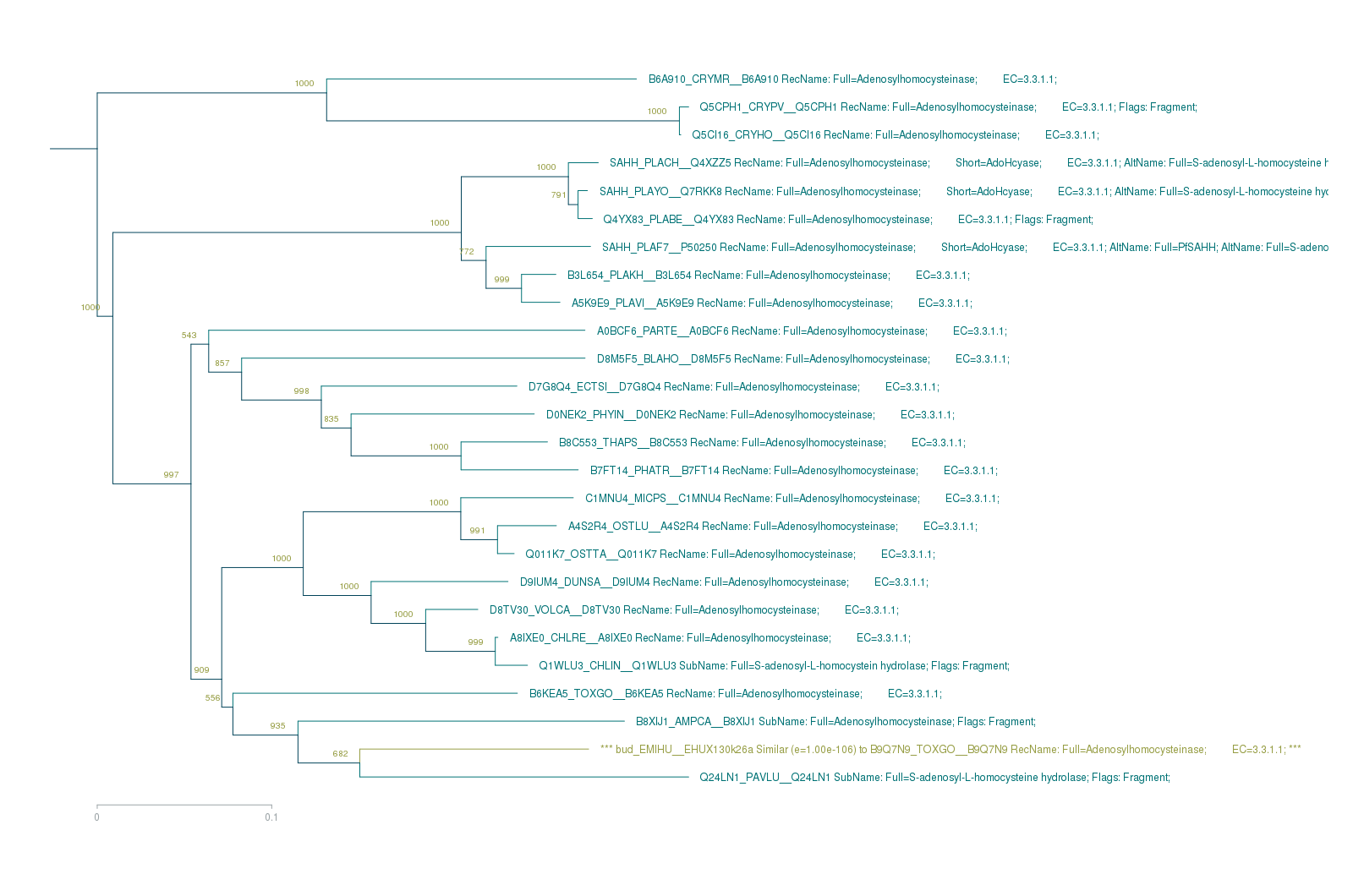

Supplement: Data S1 — Output files from BUDAPEST analysis. (ZIP) [file pone.0061868.s001.zip › BUDAPEST data/Jones_et_al_2012_Data/EHUX130k26a.png]

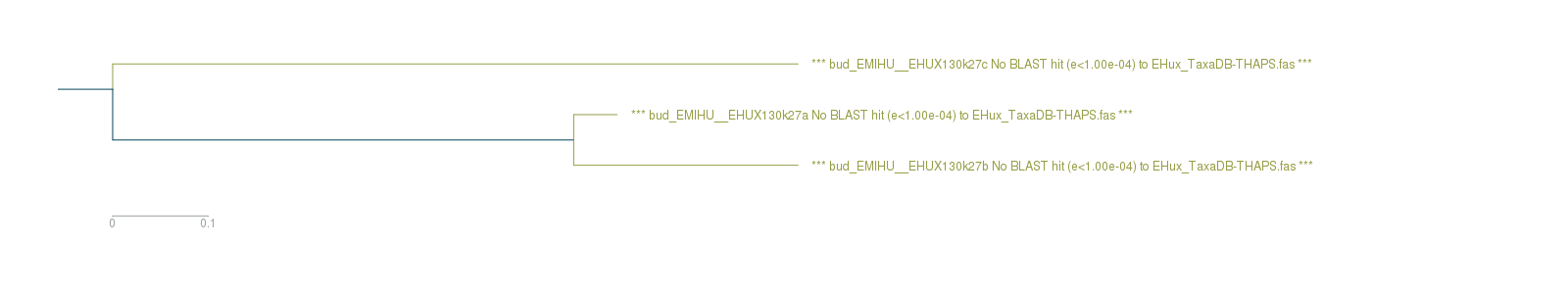

Supplement: Data S1 — Output files from BUDAPEST analysis. (ZIP) [file pone.0061868.s001.zip › BUDAPEST data/Jones_et_al_2012_Data/EHUX130k27a.png]

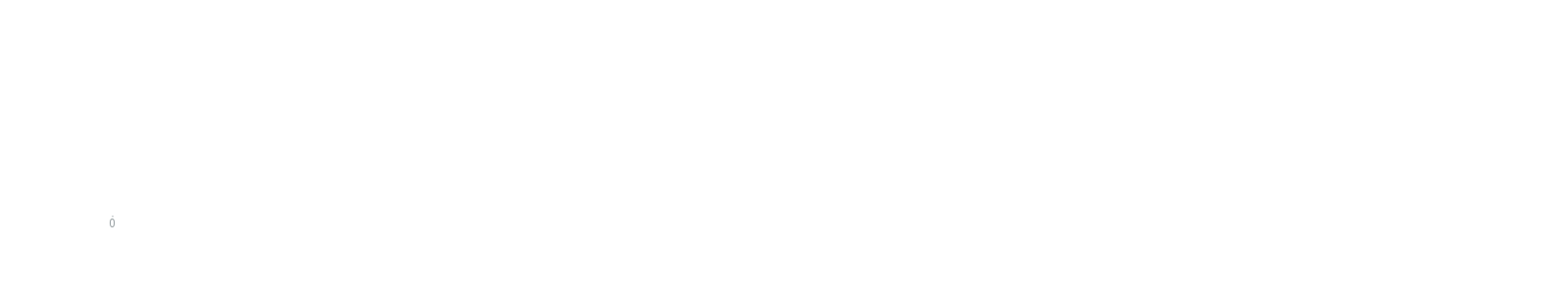

Supplement: Data S1 — Output files from BUDAPEST analysis. (ZIP) [file pone.0061868.s001.zip › BUDAPEST data/Jones_et_al_2012_Data/EHUX130k27b.png]

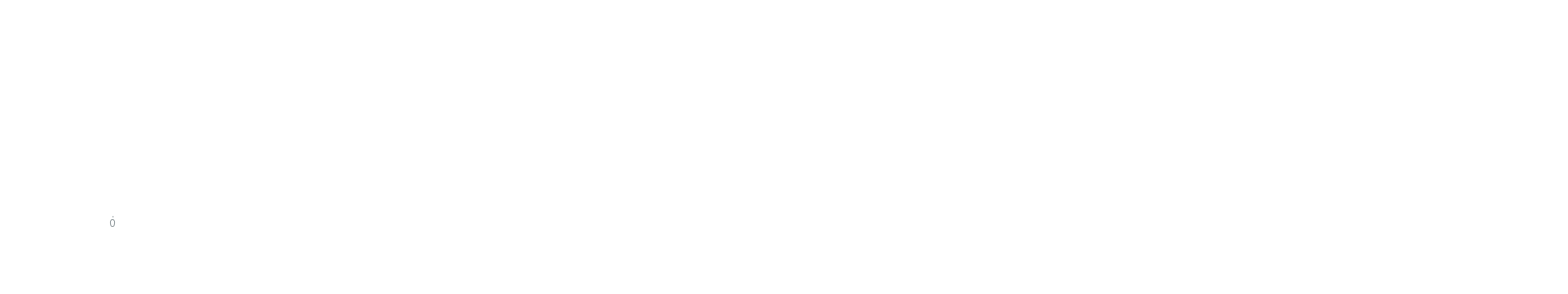

Supplement: Data S1 — Output files from BUDAPEST analysis. (ZIP) [file pone.0061868.s001.zip › BUDAPEST data/Jones_et_al_2012_Data/EHUX130k27c.png]

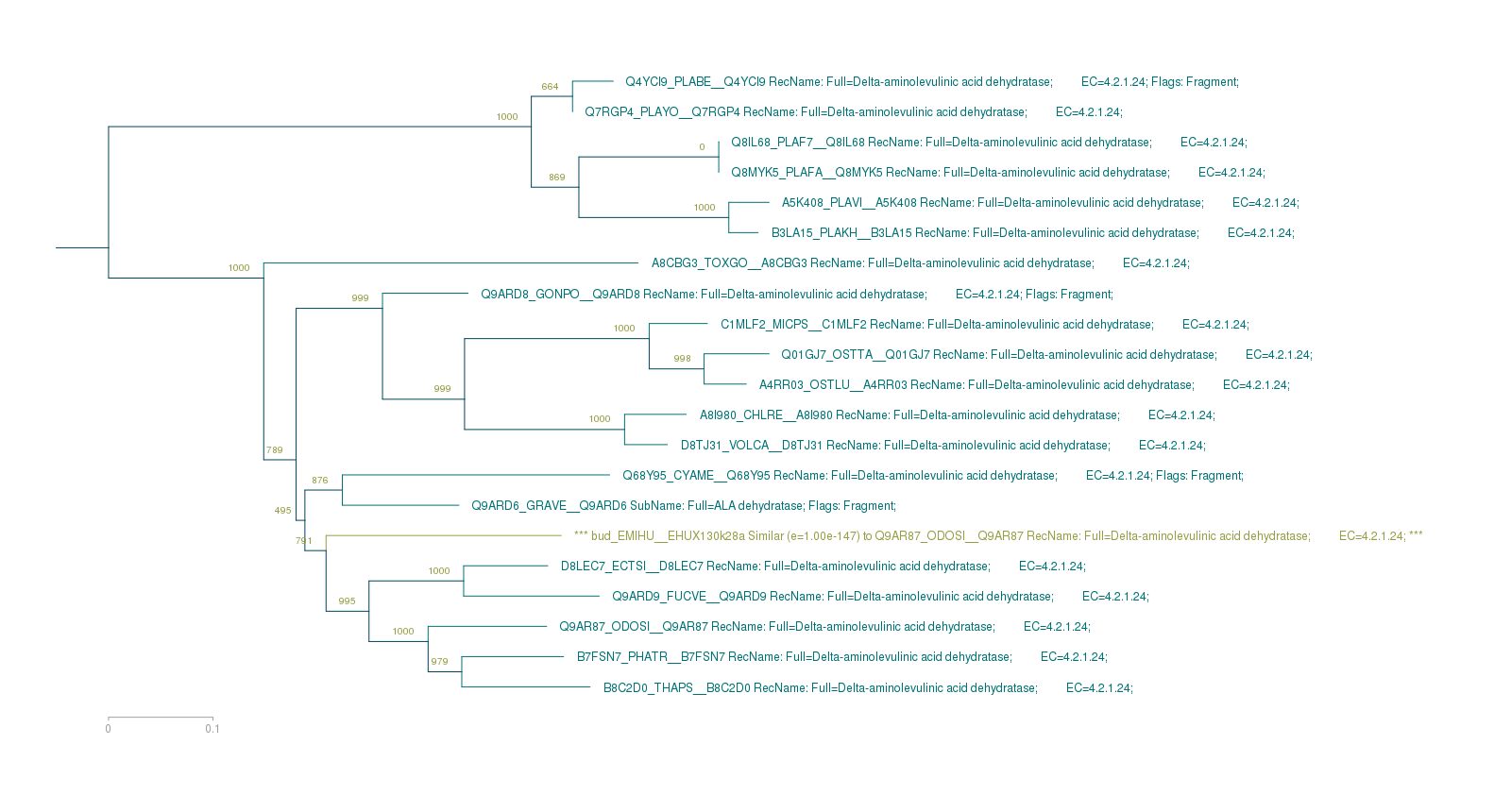

Supplement: Data S1 — Output files from BUDAPEST analysis. (ZIP) [file pone.0061868.s001.zip › BUDAPEST data/Jones_et_al_2012_Data/EHUX130k28a.png]

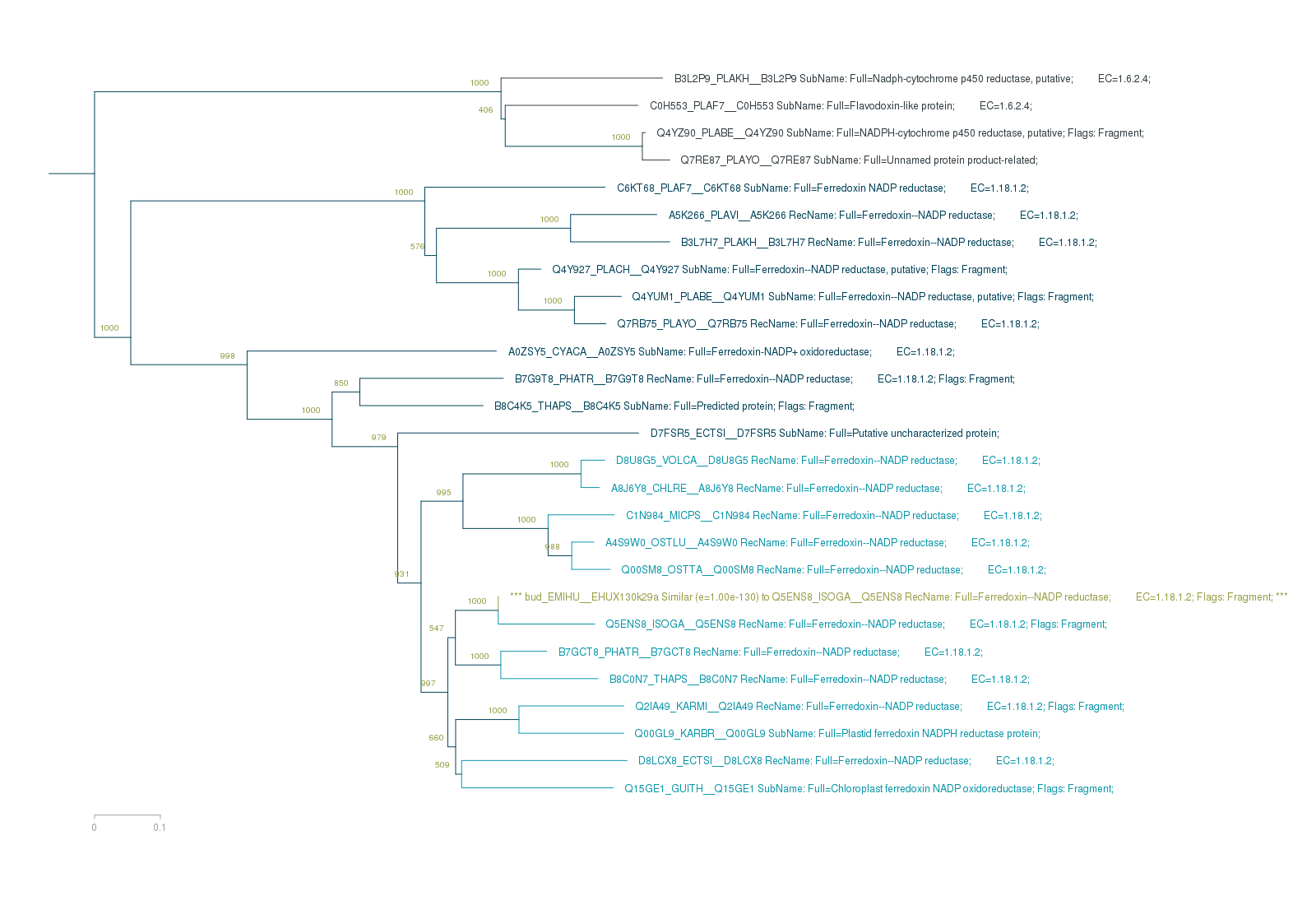

Supplement: Data S1 — Output files from BUDAPEST analysis. (ZIP) [file pone.0061868.s001.zip › BUDAPEST data/Jones_et_al_2012_Data/EHUX130k29a.png]

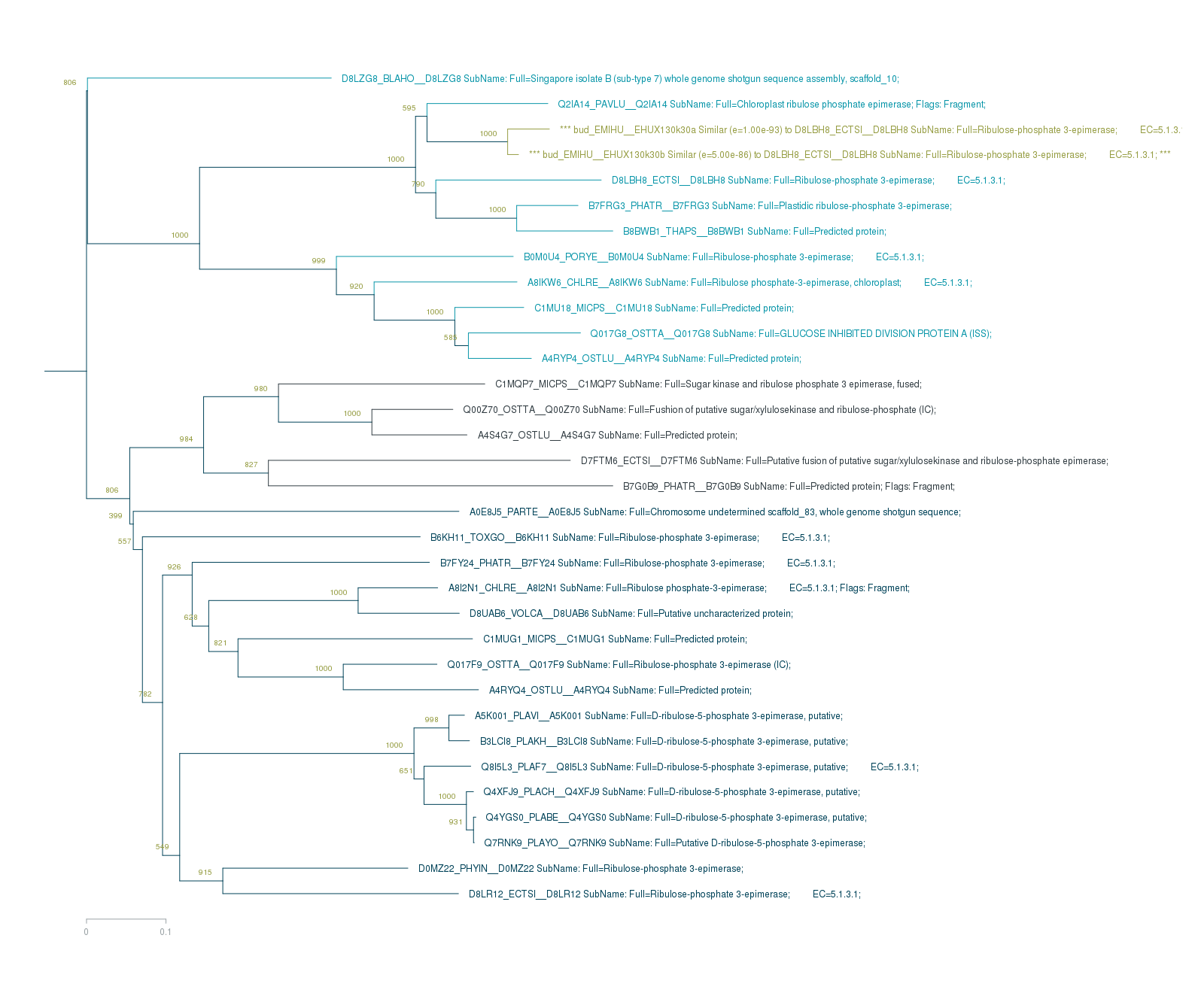

Supplement: Data S1 — Output files from BUDAPEST analysis. (ZIP) [file pone.0061868.s001.zip › BUDAPEST data/Jones_et_al_2012_Data/EHUX130k30a.png]

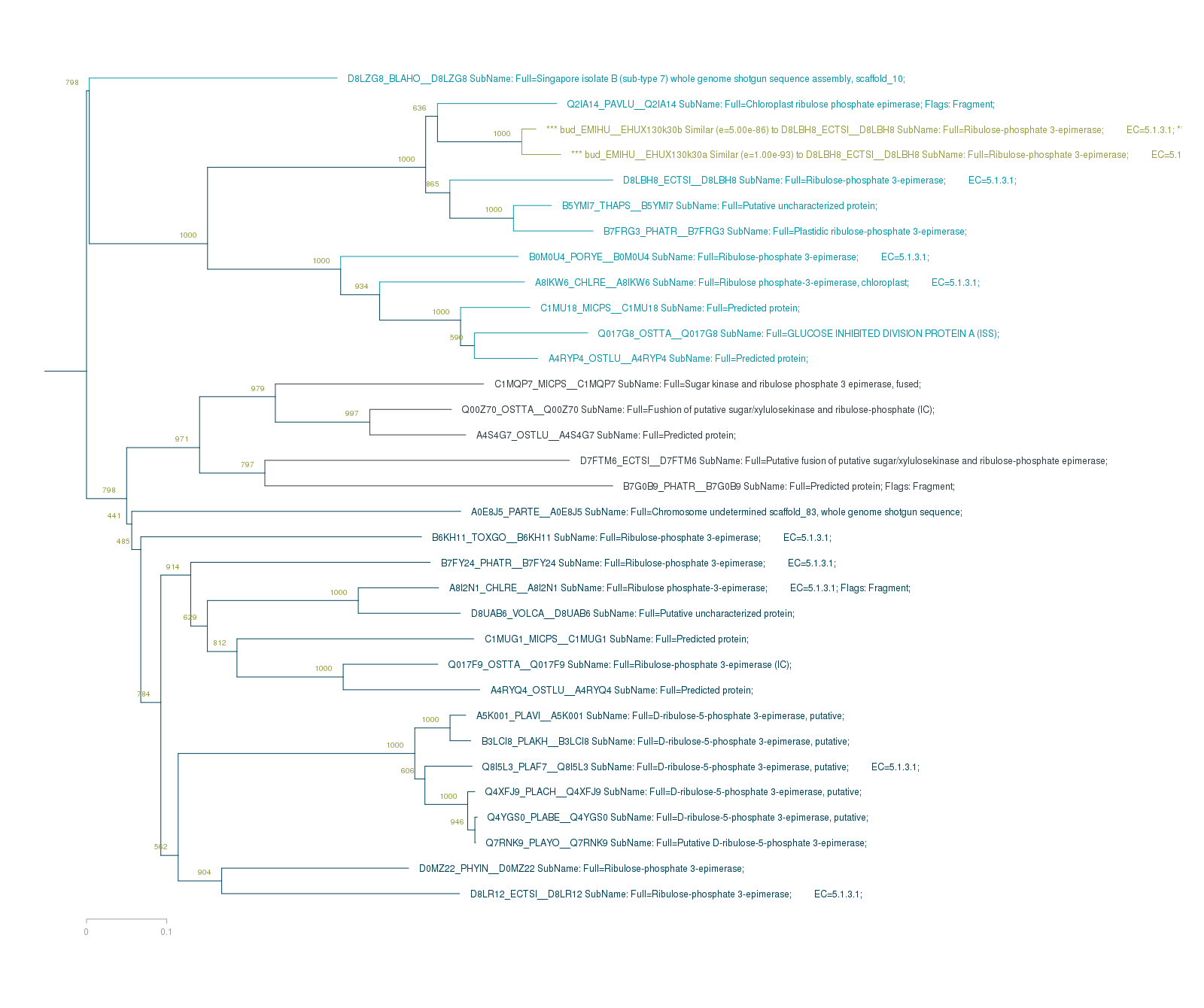

Supplement: Data S1 — Output files from BUDAPEST analysis. (ZIP) [file pone.0061868.s001.zip › BUDAPEST data/Jones_et_al_2012_Data/EHUX130k30b.png]

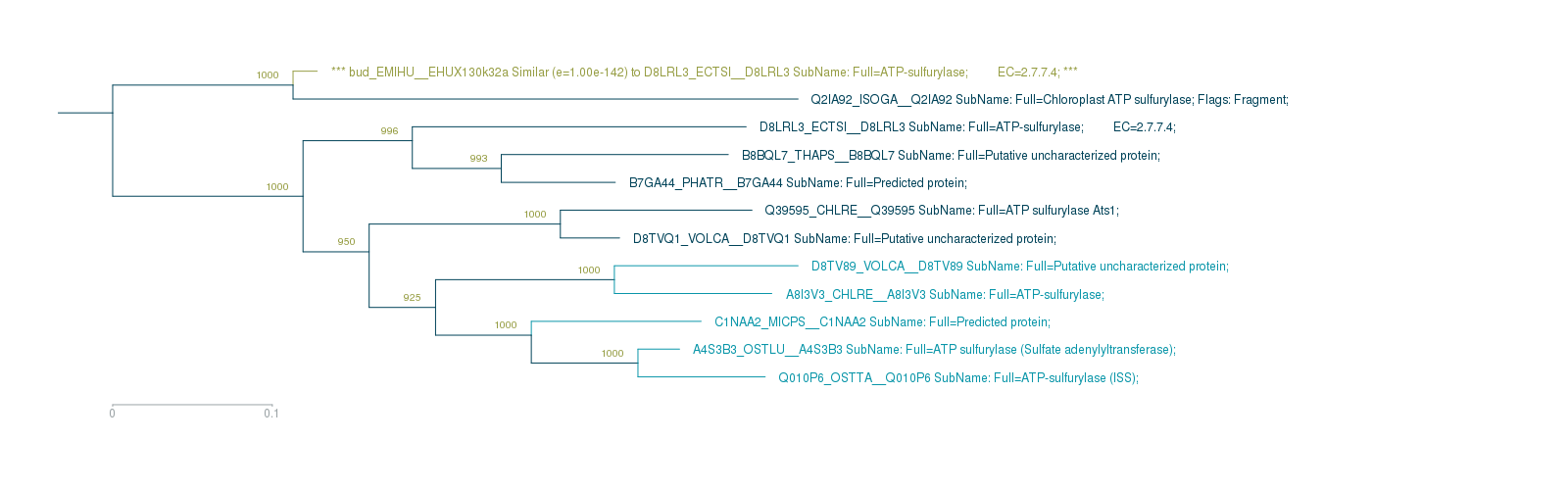

Supplement: Data S1 — Output files from BUDAPEST analysis. (ZIP) [file pone.0061868.s001.zip › BUDAPEST data/Jones_et_al_2012_Data/EHUX130k32a.png]

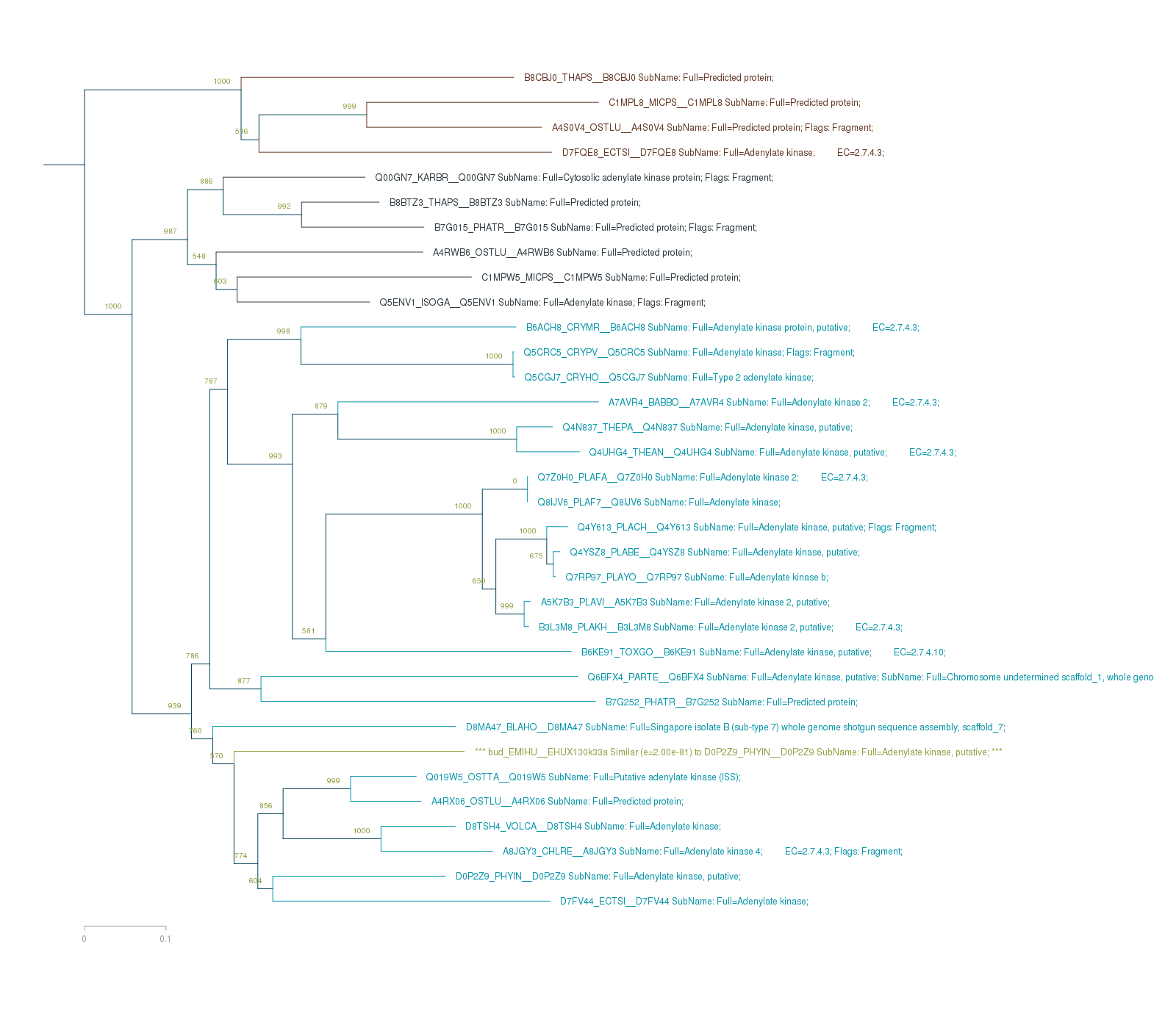

Supplement: Data S1 — Output files from BUDAPEST analysis. (ZIP) [file pone.0061868.s001.zip › BUDAPEST data/Jones_et_al_2012_Data/EHUX130k33a.png]
