# Supplementary material for: Responses of the Emiliania huxleyi Proteome to Ocean Acidification
Source: PLoS One. 2013 Apr 12;8(4):e61868. doi: 10.1371/journal.pone.0061868 (PMC3625171; doi:10.1371/journal.pone.0061868)
Supplement: Methods S1 — UPLC and MS/MS parameters; Mascot protein identification search parameters. (DOCX) [file pone.0061868.s003.docx]

Supporting information.

Methods S1.

*2D-UPLC and MS/MS*

For the first dimension separation, a 2.0 µL aliquot of the prepared protein lysates containing 3.2 µg of iTRAQ-labeled peptides was injected onto a 5 µm Xbridge BEH130 C18, 300 µm ID x 50 mm (Waters) column equilibrated in 20 mM ammonium formate, pH 10 (buffer A). The first dimension separation was achieved by increasing the concentration of acetonitrile (buffer B) in 7 steps consisting of 11, 14, 16, 20, 25, 50 and 65%. At each step, the programmed percentage composition was held for 1 min at a low rate of 1000 nL/min and the eluant diluted by H_2_O + 0.1% formic acid (buffer C) from the second dimension pump at a flow rate of 20 µL/min, effectively diluting the ammonium formate and acetonitrile, allowing trapping of the eluting peptides onto a Symmetry C18, 180 µm x 20 mm trapping cartridge (Waters, U.K.). After 20 min washing of the trap column, peptides were separated using an in-line second dimension analytical separation performed on a 75 µm ID x 200 mm, 1.7 µm BEH130 C18, column (Waters, U.K.) using a linear gradient of 5 to 40% B (buffer A = water containing 0.1% formic acid, buffer B = 95% acetonitrile containing 0.1% formic acid) over 120 min with a wash to 85% B at a flow rate of 300 nL/min. All separations were automated and performed on-line to the MS.

*Mascot protein identification search parameters*

Search parameters included a maximum of one missed cleavage for tryptic digestion and fixed modifications for methyl methane-thiosulphonation of cysteine and the N-terminus and lysine side chains using the 8-plex iTRAQ label. Variable modification for the oxidation of methionine and iTRAQ modification of tyrosine were also allowed. Precursor ion and sequence ion mass tolerances were set at 100 ppm and 0.3 Da respectively. Protein identifications required the assignment of ≥ 2 different peptides with different sequences and a significance threshold for accepting a match of p < 0.05.
