# Supplementary material for: Responses of the Emiliania huxleyi Proteome to Ocean Acidification
Source: PLoS One. 2013 Apr 12;8(4):e61868. doi: 10.1371/journal.pone.0061868 (PMC3625171; doi:10.1371/journal.pone.0061868)
Supplement: Table S1 — Mean carbonate chemistry parameters of 14.7L acclimation cultures before cell addition. (DOCX) [file pone.0061868.s004.docx]

Supporting information.

Table S1.

Mean carbonate chemistry parameters of 14.7L acclimation cultures before cell addition.

|  | Ambient | 1340 p.p.m.v. CO_2_ |
| --- | --- | --- |
| *p*CO_2_ (p.p.m.v.) | 422.8 | 1393.1 |
| [CO_2_] (µmol kg SW^-1^) | 14.1 | 46.6 |
| [CO_3_^2-^] (µmol kg SW^-1^) | 169.2 | 66.6 |
| [HCO_3_^-^] (µmol kg SW^-1^) | 1865.6 | 2132.2 |
| [DIC] (µmol kg SW^-1^) | 2048.9 | 2245.4 |
| Ω-cal | 4.03 | 1.59 |
| pH | 7.93 | 7.48 |
| TA (µmol kg SW^-1^) | 2290.3 | 2302.3 |
