# Supplementary material for: Responses of the Emiliania huxleyi Proteome to Ocean Acidification
Source: PLoS One. 2013 Apr 12;8(4):e61868. doi: 10.1371/journal.pone.0061868 (PMC3625171; doi:10.1371/journal.pone.0061868)
Supplement: Table S2 — Mean carbonate chemistry parameters associated with 14.7L acclimation cultures at t1 . (DOCX) [file pone.0061868.s005.docx]

Supporting information.

Table S2.

Mean carbonate chemistry parameters associated with 14.7L acclimation cultures at *t1*.

|  | Ambient | 1340 p.p.m.v. CO_2_ |
| --- | --- | --- |
| *p*CO_2_ (p.p.m.v.) | 452.2 | 1551 |
| [CO_2_] (µmol kg SW^-1^) | 15.1 | 52.2 |
| [CO_3_^2-^] (µmol kg SW^-1^) | 152.8 | 54.7 |
| [HCO_3_^-^] (µmol kg SW^-1^) | 1822.3 | 2032.7 |
| [DIC] (µmol kg SW^-1^) | 1990.2 | 2139.6 |
| Ω-cal | 3.62 | 1.31 |
| pH | 7.89 | 7.42 |
| TA (µmol kg SW^-1^) | 2209.1 | 2172.8 |
