# Supplementary material for: Responses of the Emiliania huxleyi Proteome to Ocean Acidification
Source: PLoS One. 2013 Apr 12;8(4):e61868. doi: 10.1371/journal.pone.0061868 (PMC3625171; doi:10.1371/journal.pone.0061868)
Supplement: Table S3 — Nitrate, phosphate and silicate information for cells at t1 . (DOCX) [file pone.0061868.s006.docx]

Supporting information.

Table S3. Nitrate, phosphate and silicate information for cells at *t1.*

| Treatment | Average phosphate (μmol kg SW^-1^) before cell addition | Average phosphate (μmol kg SW^-1^) at *t1* | % reduction during period of cell growth |
| --- | --- | --- | --- |
| 395-1 | 5.33 | 4.23 | 20.64 |
| 395-2 | 5.72 | 4.32 | 24.48 |
| 395-3 | 4.9 | 4.19 | 14.49 |
|  | Average nitrate (μmol kg SW^-1^) before cell addition | Average nitrate (μmol kg SW^-1^) at *t1* | % reduction during period of cell growth |
| 395-1 | 122.86 | 111.18 | 9.51 |
| 395-2 | 122.8 | 109.27 | 11.01 |
| 395-3 | 106.9 | 103.56 | 3.12 |
|  | Average silicate (μmol kg SW^-1^) before cell addition | Average silicate (μmol kg SW^-1^) at *t1* | % reduction during period of cell growth |
| 395-1 | 1.15 | 1.06 | 7.83 |
| 395-2 | 1.06 | 0.95 | 10.38 |
| 395-3 | 1 | 0.87 | 13 |
|  | Average phosphate (μmol kg SW^-1^) before cell addition | Average phosphate (μmol kg SW^-1^) at *t1* | % reduction during period of cell growth |
| 1340-1 | 5.99 | 4.12 | 31.22 |
| 1340-2 | 5.71 | 3.49 | 38.88 |
| 1340-3 | 5.34 | 3.38 | 36.7 |
|  | Average nitrate (μmol kg SW^-1^) before cell addition | Average nitrate (μmol kg SW^-1^) at *t1* | % reduction during period of cell growth |
| 1340-1 | 121.75 | 116 | 4.72 |
| 1340-2 | 99.63 | 89.15 | 10.51 |
| 1340-3 | 108.23 | 94.28 | 12.89 |
|  | Average silicate (μmol kg SW^-1^) before cell addition | Average silicate (μmol kg SW^-1^) at *t1* | % reduction during period of cell growth |
| 1340-1 | 1.31 | 1.16 | 11.45 |
| 1340-2 | 0.96 | 0.94 | 2.08 |
| 1340-3 | 1.22 | 1.13 | 7.38 |
