# Supplementary material for: Responses of the Emiliania huxleyi Proteome to Ocean Acidification
Source: PLoS One. 2013 Apr 12;8(4):e61868. doi: 10.1371/journal.pone.0061868 (PMC3625171; doi:10.1371/journal.pone.0061868)
Supplement: Table S4 — Nitrate, phosphate and silicate information for cells at t2 . (DOCX) [file pone.0061868.s007.docx]

Supporting information.

Table S4. Nitrate, phosphate and silicate information for cells at *t2.*

| Treatment | Average phosphate (μmol kg SW^-1^) before cell addition | Average phosphate (μmol kg SW^-1^) at *t2* | % reduction during period of cell growth |
| --- | --- | --- | --- |
| 395-1 | 6.53 | 4.14 | 36.6 |
| 395-2 | 5.65 | 4.13 | 26.9 |
| 395-3 | 5.6 | 4.21 | 24.82 |
|  | Average nitrate (μmol kg SW^-1^) before cell addition | Average nitrate (μmol kg SW^-1^) at *t2* | % reduction during period of cell growth |
| 395-1 | 121.11 | 88.76 | 26.71 |
| 395-2 | 120.96 | 99.41 | 17.82 |
| 395-3 | 125.62 | 94.13 | 25.07 |
|  | Average silicate (μmol kg SW^-1^) before cell addition | Average silicate (μmol kg SW^-1^) at *t2* | % reduction during period of cell growth |
| 395-1 | 1.04 | 0.72 | 30.77 |
| 395-2 | 1.06 | 0.84 | 20.75 |
| 395-3 | 0.93 | 0.72 | 22.58 |
|  | Average phosphate (μmol kg SW^-1^) before cell addition | Average phosphate (μmol kg SW^-1^) at *t2* | % reduction during period of cell growth |
| 1340-1 | 6.87 | 3.46 | 49.64 |
| 1340-2 | 5.86 | 3.23 | 44.88 |
| 1340-3 | 6.08 | 4.21 | 30.76 |
|  | Average nitrate (μmol kg SW^-1^) before cell addition | Average nitrate (μmol kg SW^-1^) at *t2* | % reduction during period of cell growth |
| 1340-1 | 123.31 | 97.66 | 20.8 |
| 1340-2 | 124.53 | 86.18 | 30.8 |
| 1340-3 | 127.15 | 103.79 | 18.37 |
|  | Average silicate (μmol kg SW^-1^) before cell addition | Average silicate (μmol kg SW^-1^) at *t2* | % reduction during period of cell growth |
| 1340-1 | 1.17 | 1.06 | 9.4 |
| 1340-2 | 1.14 | 0.85 | 25.44 |
| 1340-3 | 1.1 | 0.93 | 15.45 |
