# Supplementary material for: Responses of the Emiliania huxleyi Proteome to Ocean Acidification
Source: PLoS One. 2013 Apr 12;8(4):e61868. doi: 10.1371/journal.pone.0061868 (PMC3625171; doi:10.1371/journal.pone.0061868)
Supplement: Table S5 — Fv/Fm values of PSII at t2 . (DOCX) [file pone.0061868.s008.docx]

Supporting information.

Table S5. *Fv/Fm* values of PSII at *t2*.

| Treatment (*t2*) | *Fv/Fm* |
| --- | --- |
| 395-1 | 0.654 |
| 395-2 | 0.649 |
| 395-3 | 0.641 |
| 1340-1 | 0.662 |
| 1340-2 | 0.649 |
| 1340-3 | 0.637 |
